# Bayesian Inference of Reticulate Phylogenies under the Multispecies Network Coalescent

**DOI:** 10.1371/journal.pgen.1006006

**Published:** 2016-05-04

**Authors:** Dingqiao Wen, Yun Yu, Luay Nakhleh

**Affiliations:** 1 Computer Science, Rice University, Houston, Texas, United States of America; 2 BioSciences, Rice University, Houston, Texas, United States of America; Harvard University, UNITED STATES

## Abstract

The multispecies coalescent (MSC) is a statistical framework that models how gene genealogies grow within the branches of a species tree. The field of computational phylogenetics has witnessed an explosion in the development of methods for species tree inference under MSC, owing mainly to the accumulating evidence of incomplete lineage sorting in phylogenomic analyses. However, the evolutionary history of a set of genomes, or species, could be reticulate due to the occurrence of evolutionary processes such as hybridization or horizontal gene transfer. We report on a novel method for Bayesian inference of genome and species phylogenies under the multispecies network coalescent (MSNC). This framework models gene evolution within the branches of a phylogenetic network, thus incorporating reticulate evolutionary processes, such as hybridization, in addition to incomplete lineage sorting. As phylogenetic networks with different numbers of reticulation events correspond to points of different dimensions in the space of models, we devise a reversible-jump Markov chain Monte Carlo (RJMCMC) technique for sampling the posterior distribution of phylogenetic networks under MSNC. We implemented the methods in the publicly available, open-source software package PhyloNet and studied their performance on simulated and biological data. The work extends the reach of Bayesian inference to phylogenetic networks and enables new evolutionary analyses that account for reticulation.

## Author Summary

Trees have long formed in biology the basic structure with which to represent and understand evolutionary relationships. Mathematical models, computational methods, and software tools for inferring phylogenetic trees and studying their mathematical properties are currently the norm in biology. The availability of genomic data from closely related species, as well as from multiple individuals within species, have brought the two fields of phylogenetics and population genetics closer than ever. In particular, the last two decades have witnessed a great flourish in the development and implementation of phylogenetic methods based on the multispecies coalescent model to capture the intricate relationship between gene and genome evolution. However, when reticulation processes such as hybridization occur, the phylogenetic history is best represented by a network. In this work, we demonstrate how the multispecies coalescent model can be adapted to reticulate evolutionary histories and report on a Bayesian method for inference of such histories under this extended model. As networks subsume trees, the model and method provide a principled and unified statistical framework for inferring treelike and non-treelike evolutionary relationships.

## Introduction

**Fig 1:**
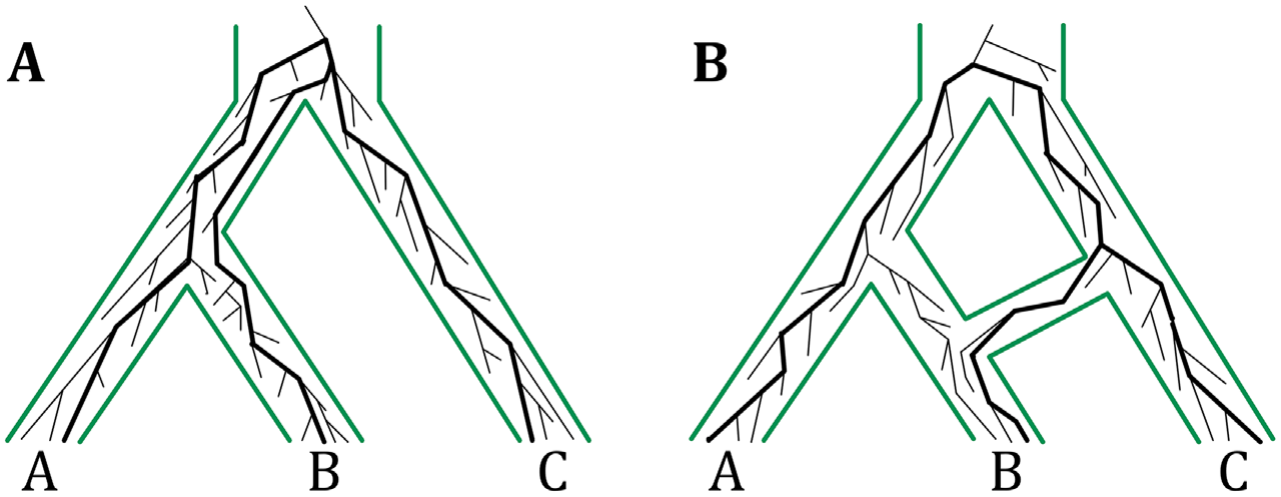
The multispecies coalescent on trees and networks. (**A**) The multispecies coalescent (MSC) links populations by a tree structure and allows for modeling gene genealogies within the branches of a species tree. The gene genealogy indicated by thick lines inside the species tree is incongruent with the species tree due to incomplete lineage sorting (ILS). (**B**) The multispecies network coalescent (MSNC) links populations by a network structure, thus allowing for reticulations events among populations. The gene genealogy indicated by thick lines inside the species network is involved in reticulation, e.g., hybridization. The gene genealogies in both panels have the same topologies, but have different probabilities under the MSC and MSNC models.

Species trees capture how species evolved and diverged from a common ancestor. These trees provide a framework for understanding how genes, genomes, and traits evolve [1, 2]. Consequently, accurate inference of species trees has been a major endeavor in evolutionary biology [3, 4]. With the availability of data from multiple genomic regions, and often whole genomes, modern inference techniques utilize all these data and employ the multispecies coalescent (MSC) model [5]. This model captures how gene (more generally, non-recombining genomic regions) genealogies grow within the branches of a species tree when extending the coalescent model [6] to multiple populations tied together by a phylogenetic tree (Fig 1**A**). MSC naturally models incomplete lineage sorting (ILS) and, when combined with models of sequence evolution, connects species trees with genomic sequence data and provides a statistical framework for species tree inference. Indeed, a wide array of methods have been devised for inferring species trees under the MSC model either directly from the sequence data [7–9] or from gene tree estimates [10, 11]; see [12–14] for recent reviews of species phylogeny inference methods.

It has long been acknowledged that the evolutionary histories of many groups of species, from across all domains of life, are reticulate. Horizontal gene transfer is ubiquitous in prokaryotic evolution [15, 16], and several bodies of work are pointing to much larger extent and role of hybridization in eukaryotic evolution than once thought [17–22]. Reticulate evolutionary histories are best modeled by *phylogenetic networks*. There are two categories of phylogenetic networks: data-display networks and evolutionary, or, explicit phylogenetic networks [23–25]. The former group is aimed at displaying pairwise relationships in the data that cannot be adequately captured by a single tree, yet not necessarily due to reticulation. The latter category provides an explicit phylogenetic model of evolutionary relationships that extends trees to allow for reticulations. The work here concerns the inference of evolutionary phylogenetic networks, or phylogenetic networks as we shall refer to them hereafter.

A phylogenetic network is a rooted, directed, acyclic graph whose leaves are labeled uniquely by a set of taxa (see Methods for a formal definition). It extends a phylogenetic tree by allowing for nodes with two parents (called *reticulation nodes*) to capture reticulation. For example, in Fig 1**B**, the phylogenetic network captures a hybridization event between species B and C. Methods for inferring phylogenetic networks with the minimum number of reticulation nodes from a set of estimated gene trees were recently introduced [26–28]. These methods assume that incongruences among gene trees are solely due to reticulation and employ parsimony as the criterion for selecting the phylogenetic networks among all possible explanations. However, as was highlighted by several recent studies [29–33], ILS could very well be at play in data sets where reticulation is suspected. Therefore, it is important to devise a statistical framework that accounts *simultaneously* for ILS and reticulation and to develop models for inference of species evolutionary histories under this framework.

Nakhleh and colleagues [30, 34, 35] recently extended the MSC model to phylogenetic networks, a model that we now call the multispecies network coalescent, or MSNC (see Methods for a formal definition). Under this model, the growth of a gene genealogy is viewed backward in time (the time flows from the root toward the leaves) within the branches of a phylogenetic network (Fig 1**B**). When a reticulation node is encountered, the genealogy traces one of the two parental species with a certain probability that is dependent on the locus for that genealogy as well as the specific reticulation node encountered. A large divergence time between C and the MRCA of A and B or a small population size of the MRCA of A and B would be unlikely to give rise to the indicated gene genealogy. However, these same settings coupled with a scenario of hybridization between B and C could very well give rise to the same gene genealogy. Yu *et al.* [30] recently devised a local search heuristic for inferring phylogenetic networks under the MSNC model. The method’s good results notwithstanding, the analyses highlighted three major issues. First, knowledge about reticulation could not be readily incorporated into the likelihood model. Second, avoiding overfitting by extra reticulations needed to be handled in a principled way. Third, a point estimate of the maximum likelihood phylogenetic network was not adequate given the closeness in likelihood of other phylogenetic networks.

To address all three issues, we devise a Bayesian framework under the MSNC model and a Markov chain Monte Carlo (MCMC) sampler of the posterior distribution on phylogenetic networks. This framework allows for systematically incorporating knowledge about reticulations and penalizing for model complexity via appropriate prior distributions. Further, the MCMC technique allows for obtaining a sample of the posterior distribution of phylogenetic networks, rather than a point estimate. Phylogenetic networks on the same taxa yet with different numbers of reticulations correspond to different numbers of parameters. Thus, walking the space of phylogenetic networks is trans-dimensional, where the number of dimensions when a new sample is proposed could decrease (due to the removal of a reticulation), increase (due to the addition of a reticulation), or remain unchanged. To account of this issue, posterior sampling is done via reversible-jump MCMC, or RJMCMC [36].

While our Bayesian framework makes use of the likelihood functions that we had derived earlier [30, 34, 35, 37], our derivations for the RJMCMC here are inspired by two works. Some of the specifics of our RJMCMC implementation are inspired by the work of Lewis *et al.* [38], where RJMCMC was employed to walk the space of phylogenetic networks with and without polytomies. For the prior on the phylogenetic network topology, our derivation was inspired by the work of Bloomquist and Suchard [39]. However, our work differs significantly from these two works in that the work of [38] is focused on trees and does not handle networks, and the work of [39] is not based on the multispecies coalescent.

We have implemented our methods in the PhyloNet software package [40], which is publicly available in open source. We tested the accuracy of the method on several simulation data sets, where we varied the topology and branch lengths of the phylogenetic network, the amount of data used in the sampling, and the prior. Our results demonstrate a good performance of the method, including the desirable property that the prior has less of an effect as the amount of data increases. We then analyzed three biological data sets: The bread wheat data set of [29], the mosquito data set of [31], and the house mouse data set that we analyzed recently in [30]. A major difference between the house mouse data set and the other two is that the former consists of multiple individuals of the same species, whereas in the other two data sets each genome is obtained from a different species. This illustrates the applicability of our method to these two different scenarios. Nonetheless, our results demonstrate the challenges with analyzing such data, particularly in terms of detecting hybridization.

To the best of our knowledge, this is the first Bayesian approach to sampling the phylogenetic network posterior under the extended MSNC. Computationally, the major bottleneck stems from the likelihood calculations under the MSNC. The computational requirements notwithstanding, our results demonstrate that this is a very promising direction to pursue in terms of application to data analysis and development of new phylogenetic network inference methods.

## Results

### A Bayesian model of reticulate phylogenies

The data *S* = {*S*_1_, …, *S*_*m*_} consists of the sequence alignments of *m* loci that we assume to be independent and recombination-free (*S*_*i*_ is the sequence alignment that corresponds to locus *i*). Our model consists of Ψ, the phylogenetic network (topology and branch lengths), and Γ, the inheritance probabilities matrix (see Methods). The posterior of the model is then given by

$$\begin{array}{c} p\left(\Psi ,\Gamma |S\right)\propto p\left(S|\Psi ,\Gamma \right)p\left(\Psi \right)p\left(\Gamma \right)=p\left(\Psi \right)p\left(\Gamma \right)\prod_{i=1}^{m}\int_{G}p\left(S_{i}|g\right)p\left(g|\Psi ,\Gamma \right)dg \end{array}$$ (1)

where the integration is taken over all possible gene trees, *p*(*S*_*i*_|*g*) is the probability of the sequence alignment *S*_*i*_ given a particular gene tree *g* [41], and *p*(*g*|Ψ, Γ) is the density of the gene tree (topologies and branch lengths) given the model parameters [30] (see S1 Text for details of the density function).

If the gene tree estimates (or, a posterior distribution thereof) of the individual loci are also of interest, the formulation above can be modified to co-estimate gene trees, in addition, as follows:

$$\begin{array}{c} p\left(\Psi ,\Gamma ,G|S\right)\propto p\left(\Psi \right)p\left(\Gamma \right)\prod_{i=1}^{m}p\left(S_{i}|g_{i}\right)p\left(g_{i}|\Psi ,\Gamma \right) \end{array}$$ (2)

where *g*_*i*_ is the estimated gene tree for locus *i*.

Indeed, this co-estimation approach is adopted by two popular species tree inference methods, *BEAST [8] and BEST [7], with the major difference being that in these methods Ψ is a tree and Γ is therefore redundant.

Inference based on Eq (1) is computationally infeasible due to the integration over all gene trees. Even Monte Carlo integration techniques would fail at estimating the integral, except for very small data sets. While sampling gene trees, as is done in *BEAST and BEST, has been shown to yield very good estimates of species and gene trees, these methods are computationally prohibitive for large data sets. Consequently, a wide array of methods for inferring species trees from gene tree estimates, rather than sequence alignments, have been introduced. In the case of networks, maximum likelihood inference of networks that uses gene tree estimates has been shown to provide good results as well [30]. If we assume that a set *G* of gene trees has been estimated for the *m* loci, then we get

$$\begin{array}{c} p\left(\Psi ,\Gamma |G\right)\propto p\left(G|\Psi ,\Gamma \right)p\left(\Psi \right)p\left(\Gamma \right)=p\left(\Psi \right)p\left(\Gamma \right)\prod_{i=1}^{m}p\left(g_{i}|\Psi ,\Gamma \right) \end{array}$$ (3)

where *g*_*i*_ is the estimated gene tree for locus *i* (with or without branch lengths). While inference from sequences directly accounts naturally for gene tree uncertainty, one has to account for this uncertainty explicitly when using gene tree estimates. Here, we will adopt the same strategy as in [30], where for each locus *i*, a set of gene tree estimates are obtained (e.g., the set of gene trees in a bootstrap analysis or the set of gene trees obtained from sampling the posterior of trees for that locus). Furthermore, while the method is applicable to data that consist of gene tree with branch lengths, we focus here on gene tree topologies alone (see results and discussion below for more on this point). The mass function for gene tree topologies given a phylogenetic network was derived in [35, 37] and is given in S1 Text.

To fully specify the model given by Eq (1), we need two priors *p*(Ψ) and *p*(Γ). For the phylogenetic network, we define a prior that is similar to that defined on ancestral recombination graphs in [39]. Given a phylogenetic network Ψ, we denote by Ψ_*top*_, Ψ_λ_, and Ψ_*ret*_ the topology, branch lengths vector, and number of reticulation nodes, respectively, of Ψ. We have

$$\begin{array}{c} p\left(\Psi |\nu ,\delta ,\eta \right)=p\left(\Psi_{ret}|\nu \right)\times p\left(\Psi_{\lambda }|\delta \right)\times p\left(\Psi_{top}|\Psi_{ret},\Psi_{\lambda }\right). \end{array}$$ (4)

where $p\left(\Psi_{ret}|\nu \right)∼\frac{1}{T_{n,m}}\text{Poisson}\left(\nu \right)$, where *T*_*n*, *m*_ is the number of phylogenetic network topologies with *n* leaves and *m* reticulation nodes, $p\left(\Psi_{\lambda }|\delta \right)∼\text{Exp}\left(\delta \right)$, and $p\left(\Psi_{top}|\Psi_{ret},\Psi_{\lambda }\right)∼\text{Exp}\left(\eta \right)$. For the inheritance probabilities Γ, we use a uniform prior on [0, 1], though a Beta distribution would also be appropriate in general cases (see S1 Text for full details).

### A reversible-jump Markov chain Monte Carlo sampler

As computing the posterior distribution given by Eq (3) is computationally intractable, we implement an Markov chain Monte Carlo (MCMC) Metropolis-Hastings algorithm. While we introduced the inheritance probabilities Γ as one parameter per reticulation node and locus, in practice, this results in a scenario where the number of parameters grows with the number of loci. Therefore, we make the simplifying assumption that there is one inheritance probability per reticulation node that is the same across all loci. In this case, Γ is a vector of length *k*, where *k* is the number of reticulation nodes in Ψ.

The description given hereafter assumes that the gene trees in the input are given by their topologies and their branch lengths are ignored. When branch lengths of the gene trees are taken into account, they pose temporal constraints on the phylogenetic network and change the moves allowed, as well as some of the quantities computed in the algorithm below. However, it is important to note that, in practice, coalescent times tend to be underestimated and that this underestimation results in biased phylogenetic estimates when sampling of loci is increased [42]. For the three biological data sets we consider below, we computed the branch lengths of the gene trees and plotted their distributions. In agreement with [42], the estimated branch lengths are very low and would result in phylogenetic networks all of whose nodes are roughly at the same level as that of the leaves. We further discuss this issue below.

In each iteration of the sampling, a new state (Ψ_*i*_, Γ_*i*_) is proposed and either accepted or rejected according to the Metropolis-Hastings ratio *r*, which is composed of the likelihoods, priors, and Hastings ratio. When the new sample changes dimensionality with respect to the current sample (which occurs only under two moves: adding a new reticulation or removing an existing reticulation), the Jacobian is also taken into account in the ratio, which results in a reversible-jump MCMC, or RJMCMC [43]. To compute the Hastings ratio, we follow the technique of [36] and illustrated by [44] for phylogenetic trees. Using this technique, given the current state **x**, a set of random numbers **u** is generated using a probability distribution with the joint probability density *g*(**u**). A deterministic function generates the new proposed state **x**^′^ = *h*(**x**, **u**). To calculate the Hastings ratio, we need to account for the move that would reverse the effects of the move **x** → **x**^′^. To propose **x** given state **x**^′^, a new set of random numbers, **u**^′^ is generated according to a distribution with density *g*^′^(**u**^′^). Then, **x** = *h*^′^(**x**^′^, **u**^′^) where *h*^′^ is another deterministic function. Green [36] replaced the Hastings ratio by

$$\begin{array}{c} \frac{g^{\prime }\left(u^{\prime }\right)}{g(u)}\left|J\right|, \end{array}$$

where *J* is the Jacobian of the transformation from {**x**, **u**} to {**x**^′^, **u**^′^}.

**Fig 2:**
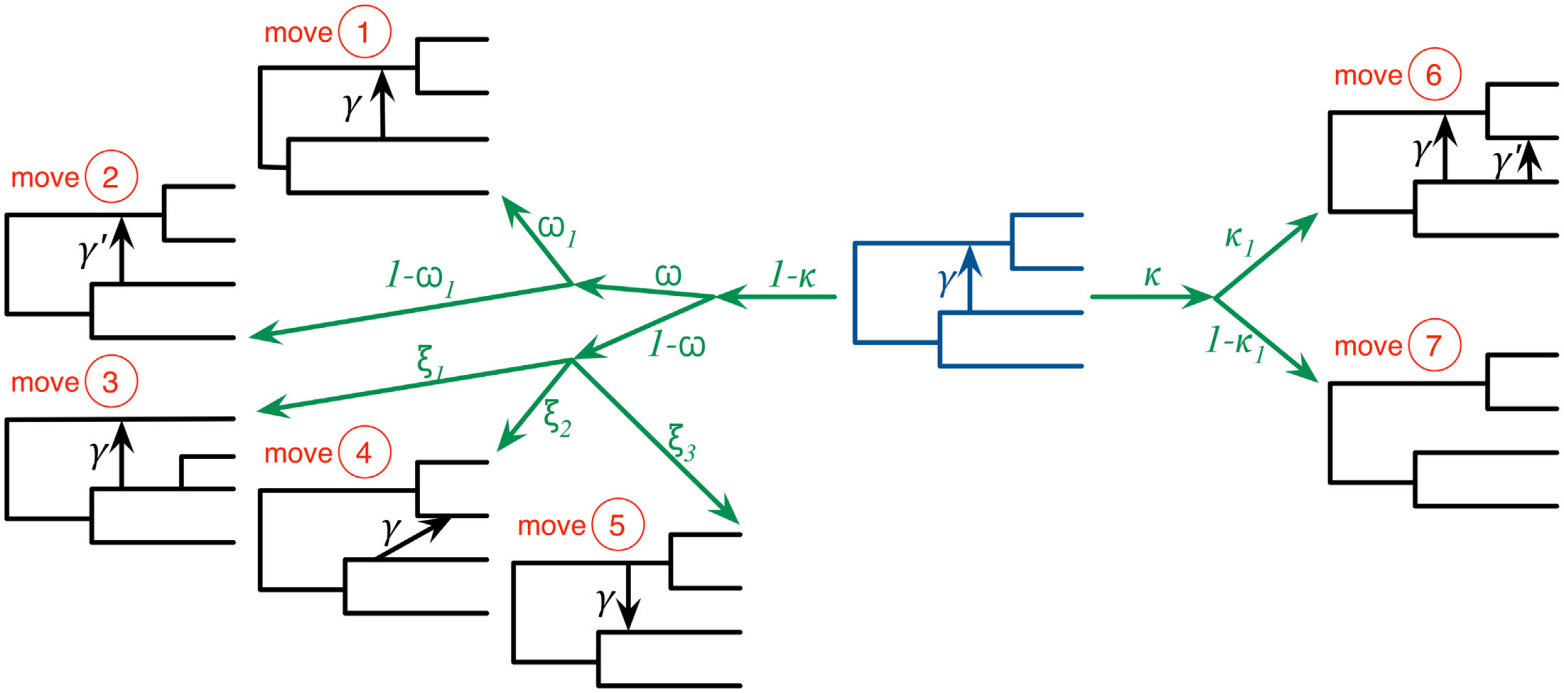
The seven moves that the MCMC sampler utilizes can be classified into ones that do not modify the topology of the phylogenetic network (moves 1 and 2), ones that modify the topology but do not change the model’s dimensions (moves 3, 4, and 5), and ones that modify the topology and model’s dimensions (moves 6 and 7). The current-state phylogenetic network is shown at the center in dark blue, and the resulting next-state phylogenetic network after each moves is show in black lines. Moves 1 and 2 modify branch lengths and inheritance probabilities, respectively. Moves 3–5 relocate one of the children of a tree node, relocate the head of a reticulation edge, and reverse the direction of a reticulation edge, respectively. Moves 6 and 7 add and remove a reticulation edge, respectively. The probabilities *κ* and *ω* determine which of the three groups of moves is selected in an iteration. Within each group, an edge is selected and a move is selected uniformly at random among all the ones that are applicable to the selected edge within that group.

Our algorithm employs seven moves to propose a new state of the Markov chain, illustrated in Fig 2 and the Methods section, along with their respective Hastings ratios whose full derivation is given in S1 Text.

We implemented our method in PhyloNet [40], which is a publicly available, open-source software package for phylogenetic network inference and analysis. We studied the performance of the method on simulated data and three biological data sets.

### Performance on simulated data

For the simulations, we used three model phylogenetic networks whose topologies and branch lengths were inspired by the estimated phylogenetic networks of the mosquito data set in [31]. Each of these networks has seven taxa, one of which is designated as an outgroup. Unfortunately, due to the prohibitive running times of computing likelihoods of networks, we currently cannot experiment with much larger (in terms of the number of taxa and/or number of reticulations) networks. The branch lengths vary from very short (about 0.5 coalescent units) to longer ones (about 1.5 coalescent units). The networks differ in the numbers of reticulations they posses (1, 2, and 3), as well as in the inheritance probabilities associated with them.

We generated gene trees for varying numbers of loci (128, 320, 800, and 2000) within each of the three networks under the multispecies coalescent process. Then, using a population mutation rate *θ* = 0.036, we simulated 1000-nucleotide sequences on the generated gene trees under the general time-reversible (GTR) model. Finally, for each generated alignment, we inferred 100 bootstrap trees under maximum likelihood and used those estimated gene trees as the data for our method. It is important to note that the estimated gene trees differed from the true gene trees on average in about 10% of their branches, with a standard deviation of about 10% as well. See S1 Text for the exact details of the model phylogenetic networks as well as the generated data.

Our results show that for the 1-reticulation model phylogenetic network, the 95% credible set consists of only one topology that is identical to the model network, regardless of the number of loci used. For the 2-reticulation model phylogenetic network, the 95% credible set on 128 and 320 loci consists of a single network that differs from the true network only in missing one of the two reticulations, whereas the 95% credible set on 800 and 2000 loci consists of the true phylogenetic network alone. For the 3-reticulation model network, using 128 and 320 loci resulted in 95% credible sets with one and two reticulations, respectively, of the true set of three reticulations. For 800 and 2000 loci, the 95% credible set consists of three different phylogenetic networks. However, these three networks are indistinguishable based on likelihood using the data, in the sense that their branch lengths and inheritance probabilities could be optimized to yield the same gene tree distributions. In this case, the differences among the topologies stem from different temporal orderings of the reticulation events involving the same pair of taxa. To summarize these results, the method performs very well in terms of recovering the true evolutionary history, including the number and placement of the reticulation events. For smaller numbers of loci, the method obtained networks that are missing one or two of the reticulations, but the rest of the evolutionary history was correct. In other words, for these smaller numbers of loci, the false positive rate was effectively 0.

In terms of runtime, the method took about 2.8 hours to run for 5 million iterations on the smallest data set (one reticulation and 128 loci) and about 9.2 hours for the same number of iterations on the largest data set (three reticulations and 2000 loci). The bottleneck in these computations comes from the likelihood calculations. The phylogenetic network topology and gene tree topology both play a role in a large variations in likelihood computation times, as reflected by large standard deviations when averaging times over the different distinct gene trees.

Full results of the performance of the method on the simulated data in terms of phylogenetic network quality and runtimes are given in S1 Text.

### Reticulate phylogenies of wheat, mosquito, and mouse genome data sets

In addition to the synthetic data, we analyzed a bread wheat genome data set from [29], a mosquito genome data set from [31], and a house mouse genome data set from [30]. It is important to note that the wheat and mosquito data sets consist of the genomes of different species, whereas the house mouse data set consists of multiple genomes from the same species. Thus, these analyses highlight the applicability of our method to the detection of intra- and inter-specific hybridization, as well as the challenges that arise in the different evolutionary scenarios.

**Fig 3:**
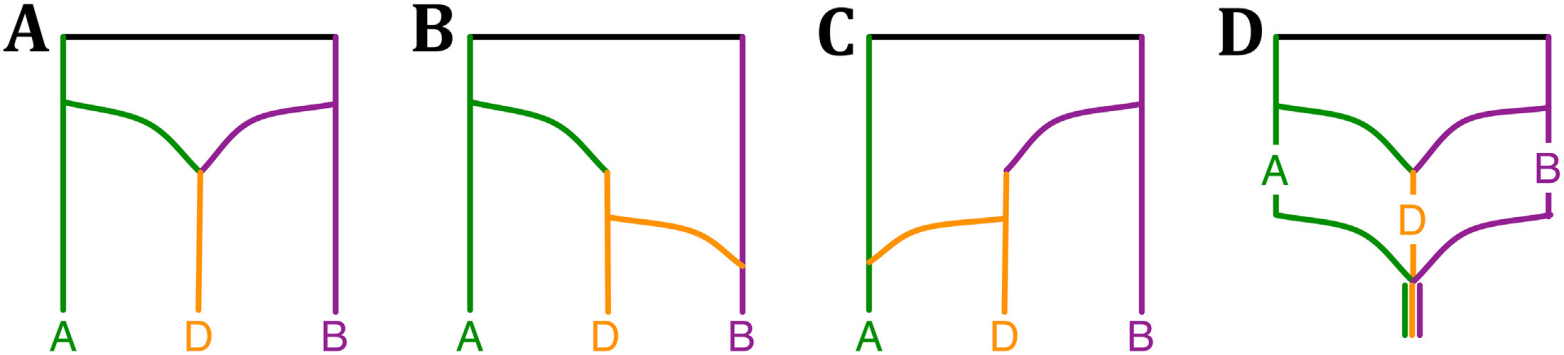
Phylogenetic history of the bread wheat. (**A**–**C**) The three phylogenetic networks that comprise the 95% credible set, (**D**) and a plausible summary of the three networks that is consistent with the model of phylogenetic history of bread wheat (Fig 3 in [29]).

The bread wheat data set consists of three subgenomes of wheat: *Triticum aestivum*, TaA (A subgenome), TaB (B subgenome) and TaD (D subgenome). Marcussen *et al.* found that each of the A and B lineages is more closely related to D than to each other, as represented by the phylogenetic network in Fig 3**A** that they inferred using the parsimony approach of [45]. Based on this network, they proposed an evolutionary history of *Triticum aestivum*, where the D genome originated from the A and B genome lineages, AABB originated from AA and BB, finally AABB and DD led to origination of AABBDD by polyploidizations and hybridizations, as shown in Fig 3 in [29].

To analyze this data set, we constructed bootstrap trees from the sequences of 2269 genes provided in [29]. The MCMC chains converged fast within a short period of burn-in, as indicated by the trace plot. The three phylogenetic network topologies in the 95% credible set are shown in Fig 3**A**–3**C**. A plausible summary of the three networks is shown in Fig 3**D**, which is consistent with the model of bread wheat proposed by Marcussen *et al.* In terms of runtime, the five million iterations of the MCMC sampling took about 2.2 hours.

While we only used gene tree topologies here, we also inferred gene trees with branch lengths under maximum likelihood. We then estimated for every pair of species the coalescence times based on all 2269 gene trees. We observed that the median pairwise distance for each pair of taxa was around 0.025, and with minimum distances of 0. Since each pairwise distance poses an upper bound on the time of the most recent common ancestor (MRCA) of that pair of species (considering the time at the leaves to be 0), then inference of phylogenetic networks using the likelihood function employed here would result in sampling only phylogenetic networks all of whose nodes have time 0. In other words, using gene tree branch lengths here would result in uninformative phylogenetic networks.

The mosquito data set [31] consists of the four autosomes and X chromosome of six species from the *An. gambiae* complex: *An. gambiae* (G), *An. coluzzii* (C), *An. arabiensis* (A), *An. quadriannulatus* (Q), *An. merus* (R) and *An. melas* (L). This data set was collected and analyzed by Fontaine *et al.* [31]. In that study, the authors inferred a species tree based on the X chromosome and postulated two major hybridization events to explain the extensive introgression. More recently, Wen *et al.* [33] reanalyzed the data set using the maximum likelihood method of [30] while restricting the number of reticulations to three, due to computational requirements. Their results corroborated parts of the evolutionary history presented in [31] and provided a different scenario for other parts.

**Fig 4:**
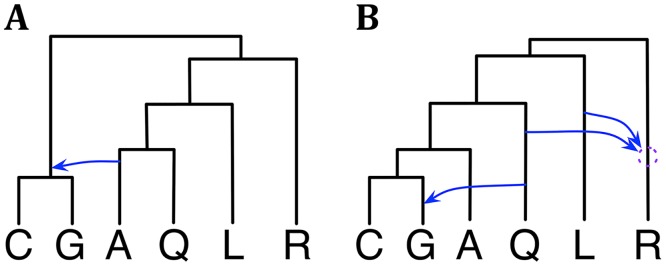
Phylogenetic history of the six mosquito genomes. (**A**) The single phylogenetic network in the 95% credible set sampled on the X chromosome data. (**B**) A summary of the three phylogenetic networks in the 95% credible set sampled on the autosome data. The dotted circle indicates the temporal order of the two reticulation events involving R cannot be discerned with confidence from the data.

We reanalyzed this data set using our Bayesian method. We first analyzed the X chromosome data alone. The 95% credible set consists of a single phylogenetic network, shown in Fig 4**A**, that agrees with [31, 33]. We then analyzed the autosome data. The 95% credible set consists of three phylogenetic networks that have the same three reticulation events but differ in terms of their temporal orders. As discussed above, these three are indistinguishable under our model, and their summary is given in Fig 4**B**. The result is in agreement with that in [33]. However, an important point here is that in this analysis, we did not bound the number of reticulations. This number was inferred as a function of the data used and the prior setting. In contrast, in [33], the number of reticulations was bounded by three, for computational reasons. In terms of runtime, this analysis took about 7.65 hours.

Given that the data set was larger than the wheat data set, we also experimented here with the prior and amount of data. In particular, we tried three values for the Poisson prior mean: 0.1, 1.0, and 10. Furthermore, while the full data set used here consists of 2791 loci, we also sampled 311 and 931 loci to create two additional data sets with smaller numbers of loci. We then conducted sampling under each combination of prior setting and data set size, our hypothesis being that as the amount of data increases, the effect of the prior setting would diminish. Indeed, we found that as the number of loci increases, the 95% credible set becomes the same regardless of the Poisson prior mean value. For the smallest data of 311 loci, a mean value of 0.1, resulted in a 95% credible set with one phylogenetic network. Yet, when the mean value was changed to 1.0 or 10, the 95% credible set contained 4 phylogenetic networks. These results further demonstrate a desirable behavior of the method.

It is worth mentioning that when setting the Poisson prior mean value to 10, the runtime increased significantly. For example, on the data set with 2791 loci, it took about 30 hours for the 5 million iterations. This is because the chain samples in this case networks with larger numbers of reticulations and whose likelihood computation time is large.

Just as in the case of the wheat data set, we also computed the pairwise distances among species based on estimated gene tree branch lengths. Once again, the results point to minimum pairwise estimates that are very close to 0 (they equal 0 for some pairs). In this case, inference the uses our likelihood formulation and gene tree branch lengths would result in an uninformative network.

Finally, the mouse data set consists of individuals sampled from five *Mus musculus* populations: two samples of *M. m. domesticus* from France (DF) and Germany (DG), and three samples of *M. m. musculus* from the Czech Republic (MZ), Kazakhstan (MK), and China (MC). For the gene genealogies, 20,639 trees were inferred for sampled loci; see [30] for details. Yu *et al.* found two main introgressions by maximum likelihood regularized by cross-validation. One involves the MRCA of {DF, DG} as a recipient population and MK, MC, or their MRCA as the donor population. The other involves MZ as a recipient population and DF, DG, or their MRCA as the donor population.

This data set differs from the previous two data sets in a significant way: The five genomes are obtained from individuals of the same species (*M. musculus*). That is, this is a data set with very low divergence. Indeed, when estimating gene trees with branch lengths, we found that all pairwise distances among the five individuals were 0; in this case, even the medians were mostly 0. Since the number of loci is very large in this case, we first analyzed the estimated gene trees for resolution. For each locus, we computed the majority-rule consensus of the 100 bootstrap trees of that locus, and counted the number of internal branches in the resulting tree. 11,457 loci had fully-resolved majority-rule consensus trees. Within this set of trees, and out of all the 105 possible binary trees on 5 taxa, 3 trees appeared with a frequency greater than 2000 each, 7 trees appeared with a frequency in the range [200, 399] each, and 11 trees appeared with a frequency in the range [50–199] each. The other 84 binary trees on 5 taxa appeared with a frequency smaller than 50 (7 trees did not appear at all in this set). In our reanalysis of this data set, we used the first set of 3 + 7 + 11 = 21 trees. In total, this amounted to using 10,575 loci.

**Fig 5:**
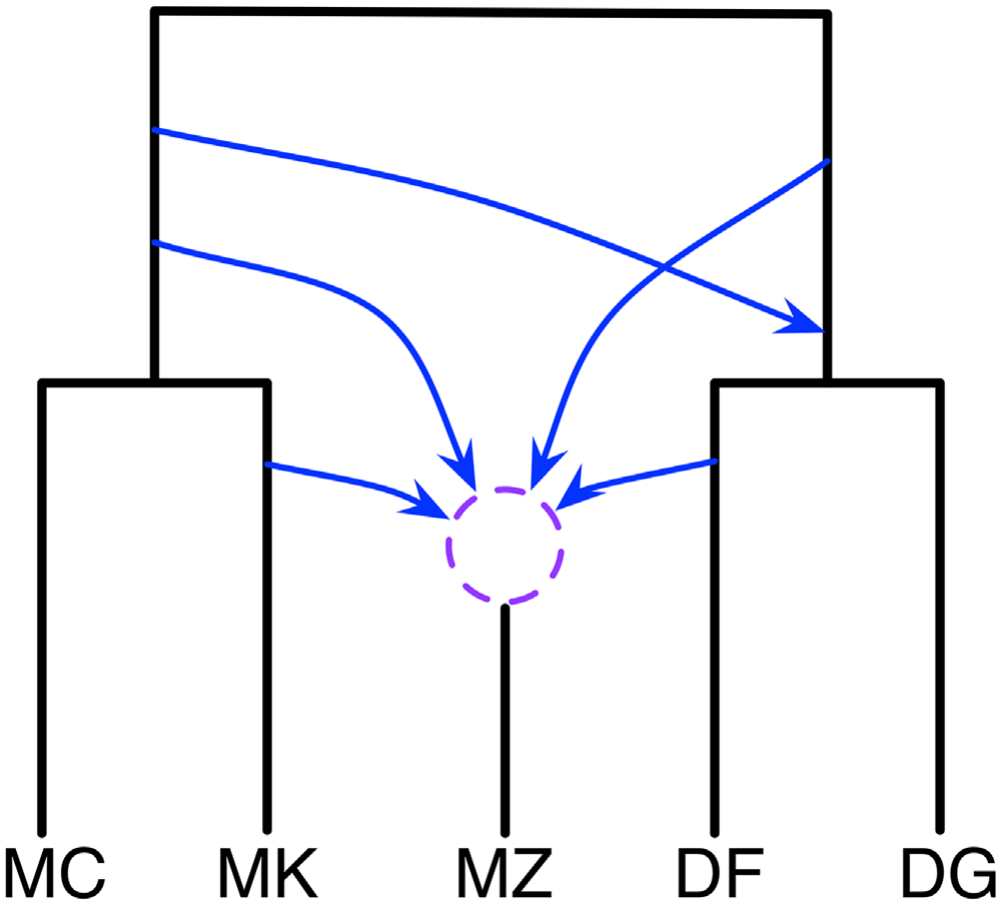
Phylogenetic history of the five mouse genomes. A summary of the six phylogenetic networks that comprise the 95% credible set. The dashed circle indicates that the different networks in the 95% credible set resolve the order of the hybridization events involving MZ in different ways.

Since the taxa correspond to individuals from the same species, more extensive gene flow is expected. Indeed, the analysis resulted in a 95% credible set with six different phylogenetic network topologies. A plausible summary of these six networks is shown in Fig 5 (the actual six networks are shown in S1 Text). This network indicates significant hybridization involving MZ. Furthermore, MC and DG are not involved in any of the hybridization events, while their ancestors and sibling taxa are involved. The network gives rise to a hypothesis of hybridization involving the ancestors of {MC,MK} and {DF, DG}, which are two different subspecies, *M. m. musculus* and *M. m. domesticus*, respectively.

In terms of computational runtime, this analysis too about 45 hours, which is much longer than the other two data sets. This is a reflection of the larger size of the data, and the complexity of the networks visited during the MCMC walk.

## Discussion

To conclude, we have devised a Bayesian framework for phylogenetic networks under the multispecies network coalescent (MSNC). In this work, the prior on the network topology allows for controlling for overfitting, as estimated phylogenetic networks could get arbitrarily complex otherwise. To enable sampling the posterior distribution of phylogenetic networks, we devised a reversible jump Markov chain Monte Carlo (RJMCMC) Metropolis-Hastings algorithm that employs a set of moves for sampling the states of the Markov chain. Implementation of the algorithm is available in the open-source software package PhyloNet [40]. We demonstrated the utility of our method on simulated data and two biological data sets. Despite its expensive computational requirements, Bayesian inference has been used extensively in the context of phylogenetic tree inference as implemented in popular software tools and programs such as MrBayes [46], *BEAST [8], BEST [7], and SNAPP [9]. Our work provides the first framework for Bayesian inference under the MSNC. The most relevant work is that of [39]. However, that work differs from ours in several significant ways.

First, the inference was performed on an ultrametric model called ancestral recombination graphs (ARGs) and the likelihood and prior computation are different. Second, the designs of the RJMCMC, mainly the moves and hasting ratios, are very different. Third, the work here assumes independent loci and inferred gene trees (while accounting for uncertainty), where the work of Bloomquist and Suchard applies to sequences and delineates recombination breakpoints. Last but not least, an implementation of the method of [39] is not publicly available which makes it hard to assess and evaluate. In particular, we employed mixing and convergence tests here, whereas it is unclear how the method of [39] performed in terms of these criteria.

Bayesian inference, particularly in the context of sampling the posterior distribution rather than obtaining a single maximum a posteriori (MAP) estimate, has become commonplace in phylogenetics. Here, we demonstrated how to extend this framework to phylogenomic analyses that account simultaneously for reticulation and incomplete lineage sorting. While the work provides a significant step in that direction, we identify several directions for further improvements. First, the full likelihood computation is very computationally extensive. It is in fact prohibitive except for data sets with small numbers of taxa and reticulation. Pseudo-likelihood-based computation was recently introduced [47, 48]. However, phylogenetic networks are not identifiable by sets of rooted triplets; hence, networks estimated based on pseudo-likelihood might differ from the true ones. Developing efficient algorithms and techniques with theoretical guarantees for estimating the likelihood of a phylogenetic network is imperative. Second, while proper priors have been developed for species trees based on models such as the birth-death model (e.g., employed by [8]), no such priors currently exist for phylogenetic networks. The prior we introduced here is a first step, but a more principled prior that captures the growth of networks just like a birth-death model captures the growth of a tree is needed. Third, while we penalized against model complexity via a Poisson distribution on the number of reticulations, devising other regularization terms would provide an alternative approach. Last but not least, while there exist standard techniques for summarizing trees sampled from the posterior distribution, such as strict or majority-rule consensus, no methods for summarizing phylogenetic networks exist. Developing methods that summarize phylogenetic networks would have impact beyond Bayesian inference.

As we discussed above, we focused here on gene tree topologies alone. The inference method can be extended to work on gene trees with their estimated branch lengths by modifying the set of operations and using the probability density function of [30]. However, under the formulation we gave here and that in [30], the coalescence times estimated for the gene trees constrain the speciation and hybridization times associated with the nodes in the phylogenetic network. For example, if at least one gene tree gives an estimate of zero for coalescence time between A and B, then either the divergence time between A and B must be set at zero in the network, or a contemporary hybridization between these two taxa must be invoked. DeGiorgio and Degnan [42] studied the effect of branch length underestimation in gene trees and its effect on species tree inference. As the study focused on gene tree estimation independently of the species phylogeny, similar issues carry over to the domain of phylogenetic network inference. Indeed, we showed for all three biological data sets studied here that divergence times based on estimated gene tree branch lengths would be problematic in terms of the network inference.

Our work is, to our knowledge, the first Bayesian approach for phylogenetic network inference under the multispecies network coalescent. The developed method allows for sampling the posterior of phylogenetic networks from multi-locus data sets while accounting for incomplete lineage sorting and hybridization. We demonstrated through analyses of simulated and biological data sets that the method performs well in practice and that it provides a powerful analytical tool for phylogenomic analyses. In particular, as the role and extent of hybridization and subsequent introgression in eukaryotic genomes continue to be investigated, we believe our method will provide a means for such a systematic investigation.

## Materials and Methods

### Phylogenetic networks

A reticulate, i.e., non-treelike, evolutionary history that arises in the presence of processes such as hybridization and horizontal gene transfer is best represented by a phylogenetic network.

**Definition 1** *A* phylogenetic X-network, *or* X-*network for short*, Ψ *is a directed*, *acyclic graph (DAG) with V* = {*r*} ∪ *V*_*L*_ ∪ *V*_*T*_ ∪ *V*_*N*_, *where*

- *indeg*(*r*) = 0 (*r is the* *root of* Ψ);
- ∀*v* ∈ *V*_*L*_, *indeg*(*v*) = 1 *and outdeg*(*v*) = 0 (*V*_*L*_ *are the* external tree nodes, *or* leaves, *of* Ψ);
- ∀*v* ∈ *V*_*T*_, *indeg*(*v*) = 1 *and outdeg*(*v*) ≥ 2 (*V*_*T*_ *are the* internal tree nodes *of* Ψ); *and*,
- ∀*v* ∈ *V*_*N*_, *indeg*(*v*) = 2 *and outdeg*(*v*) = 1 (*V*_*N*_ *are the* reticulation nodes *of* Ψ),

*E* ⊆ *V* × *V* *are the network’s edges, including* reticulation edges *whose heads are reticulation nodes, and* tree edges *whose heads are tree nodes., and ℓ*: *V*_*L*_ → X *is the* leaf-labeling *function, which is a bijection from* *V*_*L*_ *to* X.

We use *V*(Ψ) and *E*(Ψ) to denote the set of nodes and edges of phylogenetic network Ψ respectively. In addition to the topology of a phylogenetic network Ψ, each edge *b* = (*u*, *v*) in *E*(Ψ) has a length λ_*b*_ measured in coalescent units, which is the number of generations divided by effective population size on that branch.

### The multispecies network coalescent (MSNC)

As an orthologous genomic region from a set X of species evolves within the branches of the species phylogeny of X, the genealogy of this region, also called *gene tree*, can be viewed as a discrete random variable whose values are all possible gene tree topologies on the set of genomic regions. When the gene tree branch lengths are also taken into account, the random variable becomes continuous. Yu *et al.* [35] gave the probability mass function (pmf) for this discrete random variable given the phylogenetic network Ψ and an additional parameter Γ that contains the inheritance probabilities associated with reticulation nodes, which we now describe briefly.

Let *E*_*R*_ ⊆ *E*(Ψ) be the set of reticulation edges, and *ρ* = |*E*_*R*_|, and consider a data set that consists of *m* independent loci. The inheritance probabilities are given by a *ρ* × *m* matrix Γ such that for every 1 ≤ *j* ≤ *m*:

1. Γ[*b*, *j*] ∈ [0, 1] for every *b* ∈ *E*_*R*_, and
2. Γ[*b*, *j*] + Γ[*b*^′^, *j*] = 1 for every distinct pair *b*, *b*^′^ ∈ *E*_*R*_ such that *b* and *b*^′^ are incident into the same reticulation node.

For an edge *b* incident into node *v* in Ψ, the entry Γ[*b*, *j*] denotes the probability that a sample from locus *i* tracks branch *b* when “entering” the population represented by node *v*.

The mass and density functions of gene trees given a phylogenetic network, its branch lengths, and inheritance probabilities were derived in [30, 34, 35, 37]; see S1 Text for a brief discussion of these two functions. Furthermore, Yu *et al.* discussed unidentifiability issues of phylogenetic networks and their parameters from gene tree topologies [35].

### Inference using RJMCMC

The general form of the Metropolis-Hastings algorithm that we implement is given in Algorithm 1. While the algorithm is described in a way that all accepted samples are returned, in the actual implementation all samples from a burn-in period are discarded, and only a small percentage of the samples beyond that are stored (the burn-in period and percentage of samples to store are both user-defined in our implementation).

**Algorithm 1: Metropolis-Hastings.**

**Input:** A set of gene trees *G* and the number of iterations *N*.

**Output:** A collection *S* of (Ψ, Γ) samples.

Initialize Ψ_0_, Γ_0_;

**for** *i* ← 1 to *N* **do**

  Ψ_*i*_, Γ_*i*_ ← *propose*(Ψ_*i*−1_, Γ_*i*−1_);

 **if** *proposal does not change dimensionality* **then**

  $r\leftarrow (\begin{array}{c} \text{likelihood}\\ \text{ratio} \end{array})\times (\begin{array}{c} \text{prior}\\ \text{ratio} \end{array})\times (\begin{array}{c} \text{Hastings}\\ \text{ratio} \end{array})$;

 **end**

 **else**

  $r\leftarrow (\begin{array}{c} \text{likelihood}\\ \text{ratio} \end{array})\times (\begin{array}{c} \text{prior}\\ \text{ratio} \end{array})\times (\begin{array}{c} \text{Hastings}\\ \text{ratio} \end{array})\times (\text{Jacobian})$;

 **end**

 **if** *r* < *random*(0, 1) **then**

  Ψ_*i*_ ← Ψ_*i*−1_;

  Γ_*i*_ ← Γ_*i*−1_;

 **end**

 *S* ← *S* ∪ {(Ψ_*i*_, Γ_*i*_)};

**end**

**return** *S*;

The function *propose*(Ψ, Γ) proposes a sample based on the current sample and set of predefined moves that are listed in Table 1 and illustrated in Fig 2 above. As we described above, the moves might generate a phylogenetic network topology that deviates “slightly” from the conditions of Definition 1. What we mean by “slightly” is that the network could violate Definition 1 in one of the following ways:

- the proposed network topology has a cycle (moves 3–5 in Table 1 might cause this);
- the proposed network topology has two edges with the same tail and head (moves 3 and 6 in Table 1 might cause this); or,
- the proposed network has more than a single node of in-degree 0 (move 3 in Table 1 might cause this).

**Table 1:** The 7 moves that the Metropolis-Hastings algorithm employs.

1. Change-Length:	Modifies the length of a randomly selected edge
2. Change-Inheritance:	Modifies the inheritance probability of a randomly selected reticulation edge
3. Move-Tail:	Modifies the tail of a randomly selected edge whose tail is a tree node
4. Move-Head:	Modifies the head of a randomly selected edge whose head is a reticulation node
5. Flip-Reticulation:	Reverses the direction of a randomly selected reticulation edge
6. Add-Reticulation:	Adds a reticulation edge between two randomly selected edges
7. Delete-Reticulation:	Deletes a randomly selected reticulation edge

The function *propose* in Algorithm 1 selects one of these randomly and applies it to the current sample to generate a new one. Moves 1 and 2 do not change the model dimension or the network’s topology. Moves 3–5 change the network’s topology but not the model dimension. Moves 6 and 7 change the network’s topology and model dimension.

Therefore, in computing the Metropolis-Hastings ratio, our implementation explicitly tests whether the proposed network topology has any of these properties; if it does, the phylogenetic network prior is set to 0, and otherwise, the prior is set based on Eq 4.

The function *propose* selects the move as follows:

- With probability *κ*, one of the two dimension-changing moves (moves 6 and 7 in Table 1) is selected. If the current network has at least one reticulation edge, then both moves 6 and 7 are possible. Add-Reticulation is selected with probability *κ*_1_ (and Delete-Reticulation is selected with probability 1 − *κ*_1_). If the current network has no reticulation edges (i.e., it is a tree), then Add-Reticulation is selected.
- With probability 1 − *κ*, a non-dimension-changing move (moves 1–5 in Table 1) is selected.
  - With probability *ω* a non-topology-changing move (moves 1 and 2 in Table 1) is selected. If the current network has at least one reticulation edge, then Change-Length is selected with probability *ω*_1_ and Change-Inheritance is selected with probability 1 − *ω*_1_. If the current network has no reticulation edges, then Change-Length is selected.
  - With probability 1 − *ω* a topology-changing move (moves 3–5 in Table 1) is selected. Moves 3, 4, and 5 are selected with probabilities *ξ*_1_, *ξ*_2_, and *ξ*_3_, respectively, where *ξ*_1_ + *ξ*_2_ + *ξ*_3_ = 1.

Full derivation of the Hastings ratios for all seven moves is given in S1 Text.

## Supporting Information

S1 Text Supporting information file that contains method details and additional results. (PDF)
