# Correction: Bayesian Inference of Reticulate Phylogenies under the Multispecies Network Coalescent

**DOI:** 10.1371/journal.pgen.1006598

**Published:** 2017-02-08

**Authors:** Dingqiao Wen, Yun Yu, Luay Nakhleh

There is an error in the Results section, under the subsection “A Bayesian model of reticulate phylogenies” for Equation 4. Specifically, in the prior, *η* should be a hyper parameter for the prior on Ψ_*top*_ (the diameter of the reticulation events). Please view the complete, correct equation here:

$$p\left(\Psi |\nu ,\delta ,\eta \right)=p\left(\Psi_{ret}|\nu \right)\times p\left(\Psi_{\lambda }|\delta \right)\times p\left(\Psi_{top}|\Psi_{ret},\Psi_{\lambda },\eta \right).$$

Additionally, there is an error in the Results section under the subsection “A reversible-jump Markov chain Monte Carlo sampler”. Specifically, in the third paragraph, the following sentence in the MCMC algorithm is incorrect: “where *J* is the Jacobian of the transformation from {**x**, **u**} to {**x**′, **u**′}.” This sentence should read: “where |*J|* is the absolute value of the determinant of the Jacobian of the transformation from {**x**, **u**} to {**x**′, **u**′}.”
