# Supplementary material for: Bayesian Inference of Reticulate Phylogenies under the Multispecies Network Coalescent
Source: PLoS Genet. 2016 May 4;12(5):e1006006. doi: 10.1371/journal.pgen.1006006 (PMC4856265; doi:10.1371/journal.pgen.1006006)
Supplement: S1 Text — (PDF) [file pgen.1006006.s001.pdf]

Supplementary Material: Bayesian Inference of Reticulate Phylogenies Under the Multispecies Network Coalescent

Dingqiao Wen[1,*], Yun Yu[1], Luay Nakhleh[1,2,*]
1 Computer Science, Rice University, Houston, TX, USA
2 BioSciences, Rice University, Houston, TX, USA
$*$ E-mail: {dw20,nakhleh}@rice.edu.

Contents

1 A Bayesian formulation 2
1.1 The likelihood . . . 2
1.2 The pmf of gene tree topologies . . . 2
1.3 The pdf of gene trees with branch lengths . . . 2
1.4 Prior distributions . . . 3
1.5 Sampling the posterior using MCMC . . . 5
1.6 Testing the MCMC sampler . . . 8
1.7 Convergence diagnostics . . . 9

2 Simulations 10
2.1 Settings for the simulations . . . 10
2.2 Experiments and Results . . . 11

3 Analysis of a bread wheat (*Triticum aestivum*) data set 14
3.1 Data preprocessing . . . 14
3.2 MCMC settings . . . 14
3.3 Runtime. . . . 15
3.4 Results . . . 15
3.5 Gene trees with branch lengths . . . 16

4 Analysis of Anopheles mosquitoes (*An. gambiae* complex) data set 16
4.1 Data preprocessing . . . 17
4.2 MCMC settings for inference from the sex chromosome and autosomes . . . 17
4.3 Results from the X chromosome data set . . . 17
4.4 Results from the autosome data set . . . 18
4.5 Gene trees with branch lengths . . . 20

5 Analysis of a house mouse (*Mus musculus*) data set 20
5.1 Data Preprocessing . . . 21
5.2 Settings . . . 22
5.3 Results . . . 23
5.4 Gene trees with branch lengths . . . 23

1 A Bayesian formulation

1.1 The likelihood

As described in the main text, the likelihood of a phylogenetic network and inheritance probabilities is based on gene trees and assumes the trees are estimated from independent loci. The likelihood formulation and computations were derived fully in [1–3]. Since loci are assumed to be independent, the likelihood of a phylogenetic network and vector of inheritance probabilities is given in terms of the mass or density of the independent gene trees. We reproduce the probability mass function (pmf) and probability density function (pdf) of gene trees here for the sake of readability and emphasize that these functions and their computations are not a contribution of this work.

1.2 The pmf of gene tree topologies

Given a phylogenetic network Ψ, we denote by Ψ_u the set of nodes that are reachable from the root of Ψ via at least one path that goes through node $u \in V(\Psi)$. Then given a phylogenetic network Ψ and a gene tree G for some locus j, a coalescent history is a function $h : V(G) \rightarrow E(\Psi)$ such that the following two conditions hold:

- if v is a leaf in G, then $h(v) = (x, y)$ where y is the leaf in Ψ with the label of the species from which the allele labeling leaf v in G is sampled;
- if v is a node in G_u, and $h(u) = (p, q)$, then $h(v) = (x, y)$ where $y \in \Psi_q$.

Given a phylogenetic network Ψ and a gene tree G for locus j, we denote by $H_\Psi(G)$ the set of all coalescent histories of G within the branches of Ψ. Then the pmf of the gene tree is given by

$$\mathbf{P}(G|\Psi, \Gamma) = \sum_{h \in H_\Psi(G)} \mathbf{P}(h|\Psi, \Gamma), \tag{1}$$

where Γ is the inheritance probabilities matrix (see the main text) and $\mathbf{P}(h|\Psi, \Gamma)$ gives the pmf of the coalescent history random variable, which can be computed as

$$\mathbf{P}(h|\Psi, \Gamma) = \frac{w(h)}{d(h)} \prod_{b \in E(\Psi)} \frac{w_b(h)}{d_b(h)} \Gamma[b, j]^{u_b(h)} p_{u_b(h) v_b(h)}(\lambda_b). \tag{2}$$

In this equation, $u_b(h)$ and $v_b(h)$ denote the number of lineages enter and exit edge b of Ψ under coalescent history h. The term $p_{u_b(h) v_b(h)}(\lambda_b)$ is the probability of $u_b(h)$ gene lineages coalescing into $v_b(h)$ during time λ_b [**?**]. And $w_b(h)/d_b(h)$ is the proportion of all coalescent scenarios resulting from $u_b(h) - v_b(h)$ coalescent events that agree with the topology of the gene tree. This quantity without the b subscript corresponds to the root of Ψ.

1.3 The pdf of gene trees with branch lengths

We use $\tau_\Psi(v)$ to denote the height of node v in phylogeny Ψ with branch lengths λ. Given a gene tree G whose branch lengths are given by λ' and a phylogenetic network Ψ whose branch lengths are given by λ, we define a coalescent history with respect to coalescence times to be a function $h : V(G) \rightarrow E(\Psi)$, such that the following condition holds:

- for $h \in H_\Psi(G)$, if $h(v) = (x, y)$ and $\tau_\Psi(x) > \tau_G(v) \geq \tau_\Psi(y)$, then $h(v) = (x, y)$.

The quantity $\tau_G(v)$ indicates at which point of branch (x, y) coalescent event v happens. We denote the set of coalescent histories with respect to coalescence times for gene tree G and phylogenetic network Ψ by $H_\Psi(G)$. Clearly, in this case, the set H depends on λ and λ'.

Given a phylogenetic network Ψ, the pdf of the gene tree (topology and branch lengths) random variable is given by

$$p(G|\Psi, \Gamma) = \sum_{h \in H_\Psi(G)} \mathbf{P}(h|\Psi, \Gamma), \tag{3}$$

where $p(h|\Psi, \Gamma)$ gives the pdf of the coalescent history (with respect to coalescence times) random variable.

Consider a locus j, whose gene tree is G and an arbitrary $h \in H_\Psi(G)$. For an edge $b = (x, y) \in E(\Psi)$, we define $T_b(h)$ to be a vector of the elements in the set $\{\tau_G(w) : w \in h^{-1}(b)\} \cup \{\tau_\Psi(y)\}$ in increasing order. We denote by $T_b(h)[i]$ the i-th element of the vector. Furthermore, we denote by $u_b(h)$ the number of gene lineages entering edge b and $v_b(h)$ the number of gene lineages leaving edge b under h. Then we have

$$p(h|\Psi, \Gamma) = \prod_{b \in E(\Psi)} \left[\prod_{i=1}^{|T_b(h)|-1} e^{-\binom{u_b(h)-i+1}{2}(T_b(h)_{i+1} - T_b(h)_i)} \right] \times e^{-\binom{v_b(h)}{2}(\tau_\Psi(b) - T_b(h)_{|T_b(h)|})} \times \Gamma[b, j]^{u_b(h)}. \tag{4}$$

1.4 Prior distributions

Prior on the phylogenetic network. We define a prior that is similar to that defined on ancestral recombination graphs in [4]. We have

$$p(\Psi|\nu, \delta, \eta) = p(\Psi_{ret}|\nu) \times p(\Psi_\lambda|\delta) \times p(\Psi_{top}|\Psi_{ret}, \Psi_\lambda, \eta). \tag{5}$$

It is important to note here that if Ψ_{top} does not follow the phylogenetic network definition (Main Text), then $p(\Psi|\nu, \delta, \eta) = 0$. This is very important since in the MCMC kernels we describe below, we allow the moves to produce directed graphs that slightly deviate from the definition; in this case, having the prior be 0 guarantees that the proposal is rejected. Using the strategy, rather than defining only "legal" moves simplifies the calculation of the Hastings ratios. See more details below.

We assume a Poisson distribution with hyperparameter ν on the number of reticulation nodes in Ψ, weighted by 1 over the number of networks with that number of reticulations. More specifically, the Poisson prior gives a probability of $\frac{\nu^m e^{-\nu}}{m!}$ for a network having m reticulation nodes. The weight is $1/T_{n,m}$, where n is the number of leaves in the network, and $T_{n,m}$ is the number of networks that have n leaves and m reticulation. Putting these two together, for a phylogenetic network Ψ with n leaves and m reticulation nodes, we have

$$p(\Psi_{ret}|\nu) = \frac{1}{T_{n,m}} \text{Poisson}(m, \nu).$$

- For all moves except Add-Reticulation and Delete-Reticulation, the prior ratio on Ψ_{ret} between the next- and current-state networks is 1.

- For the Delete-Reticulation move, the prior ratio is

$$\frac{T_{n,m}}{T_{n,m-1}} \cdot \frac{\text{Poisson}(m-1, \nu)}{\text{Poisson}(m, \nu)}.$$

- For the Add-Reticulation move, the prior ratio is

$$\frac{T_{n,m}}{T_{n,m+1}} \cdot \frac{\text{Poisson}(m+1, \nu)}{\text{Poisson}(m, \nu)}.$$

To the best of our knowledge, there is no known closed formula or algorithm for calculating $T_{n,m}$ for general values of n and m. However, if k is the number of edges in a phylogenetic network Ψ with m reticulations, then the number of ways to add an additional reticulation edge to Ψ is bounded by $k(k-1)$. Based on this observation, we make use of the recurrence

$$T_{n,m} = T_{n,m-1} \cdot k \cdot (k-1)$$

where $k = 2(n-1) + 3(m-1)$ and $T_{n,0} = c$, where c is, in theory, the number of rooted phylogenetic networks, but in practice can be any non-zero value since in the prior ratio, this value cancels out. In our implementation, for the prior ratios of Delete-Reticulation and Add-Reticulation moves, we evaluate this recurrence explicitly for the numerator and denominator.

This prior penalizes against adding many reticulations. From a biological perspective, it is not unreasonable to have a prior belief of a small number of reticulations. From a computational perspective, computing the likelihood of a network is prohibitively slow. The computational requirements of this step are affected heavily by the number of reticulations and their configurations (that is, how they are placed in the network) [2]. Penalizing against very complex networks helps with the feasibility of these computations.

We assume an exponential distribution on the branch lengths so that every branch length is $\sim \text{Exp}(\delta)$.

While [4] assumed a uniform distribution on all topologies with the same number of reticulation nodes, a reasonable prior for phylogenetic networks is one that favors reticulations between closely related species. This would make a difference particularly in cases where the number of taxa is very large and the species form groups with small divergences within and large divergences across those groups.

Consider a phylogenetic network Ψ and inheritance probabilities Γ, and let x be a reticulation node in Ψ whose two parents in Ψ are v_1 and v_2. Let T be the tree that results from Ψ by removing every edge that has inheritance probability < 0.5 (if both edges incoming a reticulation node have inheritance probability of exactly 0.5, then both edges are removed one at a time and the one that results in the smaller diameter is chosen). Then, the diameter of x, denoted by $d(x)$, is defined as

$$d(x) = l_{r \rightsquigarrow v_1} + l_{r \rightsquigarrow v_2} + \lambda_{(v_1,x)} + \lambda_{(v_2,x)},$$

where r is the MRCA (most recent common ancestor) of v_1 and v_2 in T, $l_{r \rightsquigarrow v_1}$ and $l_{r \rightsquigarrow v_2}$ are the lengths of the paths from r to v_1 and v_2, respectively, in T, and $\lambda_{(v_1,x)}$ and $\lambda_{(v_2,x)}$ are the lengths of the two edges (v_1, x) and (v_2, x), respectively, in Ψ. A figure illustrating the measurement of diameter is shown in Fig. 1. We now assume an exponential distribution on the diameter of a reticulation nodes in Ψ, so

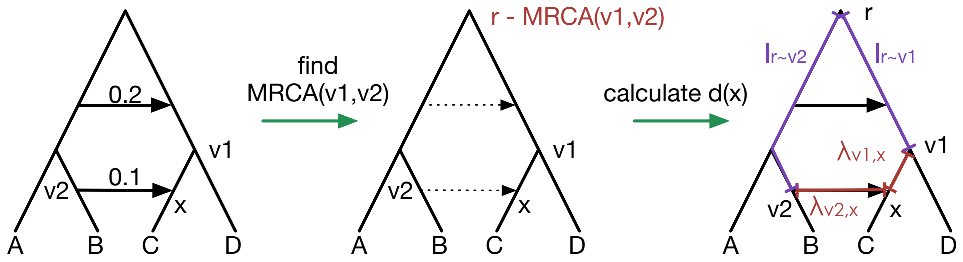

Figure 1. An illustration of the diameter of a reticulation node x.

that $d(x_i) \sim \text{Exp}(\eta)$ for each reticulation nodes x_i, and we treat the reticulation nodes in the network as independent.

In the analyses we report in the main manuscript and below, we used a uniform prior on the diameter since the number of taxa was very small and the taxa themselves are very closely related.

Prior on the inheritance probabilities. As discussed above, for each reticulation node, there are two edges incoming into it, b and b'. For every locus i, we associate values $\Gamma[b,i]$ and $\Gamma[b',i]$ such that $\Gamma[b,i]+\Gamma[b',i]=1$. We propose $\Gamma[b,i] \sim \text{Beta}(\alpha,\beta)$ for a prior. In the absence of any information on the inheritance probabilities, setting $\alpha=\beta=1$ amounts to using a uniform prior on $[0,1]$. If the amount of introgressed genomic data is suspected to be small in the genome, the hyper-parameters α and β can be appropriately set to bias the inheritance probabilities to values close to 0 and 1 (a U-shaped distribution).

1.5 Sampling the posterior using MCMC

For each of the 7 moves (see Main Text), we now describe how it is implemented and the Hastings ratio (and the Jacobian wherever relevant).

Change-Length. An edge is selected uniformly at random and the branch length ℓ of the edge is modified into ℓ' using the proposal (similar to [5])

$$\ell' = \ell e^{\sigma(u-0.5)}$$

where σ is a tuning parameter and $u \sim \text{Uniform}(0,1)$. The Hastings ratio is $\frac{\ell'}{\ell}$ (derived in [5]). It is important to note that if a single individual is sampled per species (or, per taxon that labels a leaf in the network), then modifying the length of an external branch (a branch that is incident with a leaf) does not affect the likelihood of the network. The same observation holds for the lengths of reticulation edges whose head is the parent of a leaf in the network; see Fig. 2. In this case, edges to which Change-Length

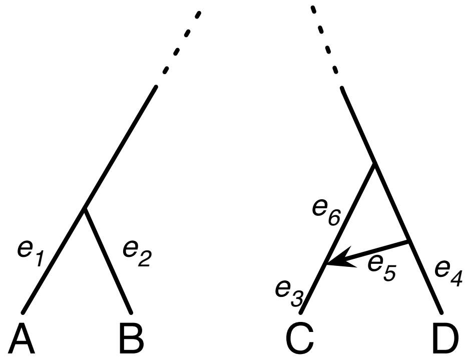

Figure 2. If a single individual is sampled from each of the four taxa A, B, C, D, then the lengths of branches $e_1,\ldots,e_6$ are not identifiable when gene tree topologies are used to infer the phylogenetic network. If, for example, two or more individuals are sampled from taxon C, then the lengths of branches e_3, e_5, and e_6 might be possible to estimate from gene tree topologies. Beyond setting the lengths of such branches immediately upon their creation during the MCMC sampling, their lengths are not sampled; that is, Change-Length is not applied to such branches, unless at least two individuals are sampled "below" the branch.

is applied exclude the external tree edges and reticulation edges whose head is the parent of a leaf.[1]

[1] Note that other branches in the network might have an unidentifiable length (alternatively, modifying their length does not affect the likelihood). Characterizing those is not a simple task, and we focus here on the two types of edges that we have listed, namely external tree branches and reticulation branches whose head is the parent of a leaf.

Change-Inheritance. A reticulation edge is selected uniformly at random from the set of all reticulation edges and the inheritance probability γ associated with it is modified into γ' using the proposal

$$\gamma' = \begin{cases} \gamma + u & \text{if} \quad 0 \leq \gamma + u \leq 1 \\ -(\gamma + u) & \text{if} \quad \gamma + u < 0 \\ 2 - (\gamma + u) & \text{if} \quad \gamma + u > 1 \end{cases}$$

where $u \sim \text{Uniform}(-0.1, +0.1)$. The value 0.1 can be replaced by a tuning parameter for a more general setting. Under this setting, the Hastings ratio is $\frac{p(\gamma|\gamma')}{p(\gamma'|\gamma)} = 1$.

Move-Tail. An edge (x, y_1) is selected uniformly at random from the set of all edges whose tail is a tree node. Let w be the parent of x (if x is the root node, then w does not exist) and y_2 be the second child of x (in addition to y_1). Let v_1 be a node such that $v_1 \notin \{w, x, y_1, y_2\}$ (in particular, v_1 could be the root if neither x nor w is the root). The following operations are performed:

1. If v_1 is not the root, let u_1 be a parent of v_1. Then,
   (a) two new edges are added: (u_1, x) and (x, v_1);
   (b) if x is not the root of the network (node w exists), then (w, y_2) is also added;
   (c) $\lambda_{(u_1,x)} + \lambda_{(x,v_1)} = \lambda_{(u_1,v_1)} = \ell_1$, $\lambda_{(u_1,x)} \sim \text{Uniform}(0, \ell_1)$;
   (d) $\lambda_{(w,y_2)} = \lambda_{(w,x)} + \lambda_{(x,y_2)} = \ell_2$ (if w exists);
   (e) If v_1 was a reticulation node before the move, then $\gamma_{(x,v_1)} = \gamma_{(u_1,v_1)}$;
   (f) If y_2 was a reticulation node before the move, then $\gamma_{(w,y_2)} = \gamma_{(x,y_2)}$; and,
   (g) Finally, delete the edges (along with their parameters): (w, x), (x, y_2), and (u_1, v_1).
2. If v_1 is the root:
   (a) two new edges are added: (x, v_1) and (w, y_2);
   (b) $\lambda_{(x,v_1)} = \ell_r = -\frac{1}{\delta}\ln(1 - w_1)$ where $w_1 \sim \text{Uniform}(0, 1)$;
   (c) $\lambda_{(w,y_2)} = \lambda_{(w,x)} + \lambda_{(x,y_2)}$;
   (d) If y_2 was a reticulation node before the move, then $\gamma_{(w,y_2)} = \gamma_{(x,y_2)}$; and,
   (e) Finally, delete the edges (along with their parameters): (w, x) and (x, y_2).

It is important to note here that we do not allow a selection where x is the root of the network and y_2 is a reticulation node whose parents are x and y_1, since in this case applying this move would result in y_2 becoming a tree node and, consequently, modify the dimension of the model. If the nodes are selected with this configuration, the move is nullified and a new proposal is made.

Hereafter, we use Δt to represent an infinitesimally small region near the proposed point in a distribution. The Hastings ratio for this move is $\frac{\Delta t \cdot 1/\ell_2}{\Delta t \cdot 1/\ell_1} = \frac{\ell_1}{\ell_2}$ when x is not the root before or after proposal, $\frac{\Delta t \cdot 1/\ell_2}{\Delta t \cdot \delta e^{-\delta \ell_r}} = \frac{1}{\ell_2 \cdot \delta e^{-\delta \ell_r}}$ when x is the root after proposal, and $\frac{\Delta t \cdot \delta e^{-\delta \ell_r}}{\Delta t \cdot 1/\ell_1} = \ell_1 \cdot \delta e^{-\delta \ell_r}$ when x is the root before proposal.

Move-Head. A reticulation edge $e = (x, y)$ is selected uniformly at random from the set of all reticulation edges. Let u_1 be the other parent of y (in addition to x) and v_1 be the child of y. The two edges (u_1, y) and (y, v_1) are deleted (along with their parameters) and replaced by a new edge $e_1 = (u_1, v_1)$ whose length is the sum of the two original lengths $\lambda_{e_1} = \lambda_{(u_1,y)} + \lambda_{(y,v_1)} = \ell_1$. Then, a new edge $e_2 = (u_2, v_2)$, with $e_2 \neq e_1$, is selected uniformly at random, deleted, and replaced by two new edges (u_2, y) and (y, v_2) whose branch lengths satisfy the conditions $\lambda_{(u_2,y)} + \lambda_{(y,v_2)} = \lambda_{(u_2,v_2)} = \ell_2$, $\lambda_{(u_2,y)} \sim \text{Uniform}(0, \ell_2)$. The length and inheritance probability of the original reticulation edge e are unchanged (and an inheritance probability of $1 - \gamma_e$ is assigned to (u_2, y)). The Hastings ratio in this case is $\frac{\Delta t \cdot 1/\ell_1}{\Delta t \cdot 1/\ell_2} = \frac{\ell_2}{\ell_1}$.

Flip-Reticulation. Let $e = (x, y)$ be the randomly selected reticulation edge. Let u_1 be the other parent of y (in addition to x) and v_1 be the child of y. Let u_2 be the parent of x and v_2 be the other child of x (in addition to y). The two edges (u_1, y) and (y, v_1) are deleted (along with their parameters) and replaced by two edges (u_1, x') and (x', v_1) under the condition that $\lambda_{(u_1,x')}+\lambda_{(x',v_1)} = \lambda_{(u_1,y)}+\lambda_{(y,v_1)} = \ell_1$, $\lambda_{(u_1,x')} \sim \text{Uniform}(0, \ell_1)$. The two edges (u_2, x) and (x, v_2) are deleted (along with their parameters) and replaced by two edges (u_2, y') and (y', v_2) under the condition that $\lambda_{(u_2,y')}+\lambda_{(y',v_2)} = \lambda_{(u_2,x)}+\lambda_{(x,v_2)} = \ell_2$, $\lambda_{(u_2,y')} \sim \text{Uniform}(0, \ell_2)$. The edge (x, y) is deleted and replaced with a new edge (x', y'). The inheritance probability of edge (u_2, y') and (x', y') are $1 - \gamma_e$ and γ_e respectively. The Hastings ratio in this case is $\frac{\Delta t \cdot 1/\ell_1 \cdot \Delta t \cdot 1/\ell_2}{\Delta t \cdot 1/\ell_2 \cdot \Delta t \cdot 1/\ell_1} = 1.0$.

Add-Reticulation. Two edges $e_1 = (u_1, v_1)$ and $e_2 = (u_2, v_2)$ are selected uniformly at random from the set of all edges in the network. Edge e_1 is replaced by two edges $e_{11} = (u_1, x_1)$ and $e_{12} = (x_1, v_1)$, where x_1 is a new node. The length

$$\lambda_{e_{11}} = \lambda_{e_1} w_1$$
$$\lambda_{e_{12}} = \lambda_{e_1}(1 - w_1)$$

where $w_1 \sim \text{Uniform}(0, 1)$. Similarly, edge e_2 is replaced by two edges $e_{21} = (u_2, x_2)$ and $e_{22} = (x_2, v_2)$, where x_2 is a new node. The length

$$\lambda_{e_{21}} = \lambda_{e_2} w_2$$
$$\lambda_{e_{22}} = \lambda_{e_2}(1 - w_2)$$

where $w_2 \sim \text{Uniform}(0, 1)$. Finally, a new edge $e_r = (x_1, x_2)$ is added with length

$$\ell_r = -\frac{1}{\delta} \ln(1 - w_3)$$

where $w_3 \sim \text{Uniform}(0, 1)$ and inheritance probability

$$\gamma_r = w_4$$

where $w_4 \sim \text{Uniform}(0, 1)$ (in which case the inheritance probability of edge (u_2, x_2) is set to $1 - \gamma_{e_r}$).

Since removing this reticulation edge does not require setting a length and inheritance probability, the Jacobian in this case involves:

- $\frac{\partial \lambda_{e_{11}}}{\partial \lambda_{e_1}} = w_1$, $\frac{\partial \lambda_{e_{11}}}{\partial w_1} = \lambda_{e_1}$, $\frac{\partial \lambda_{e_{12}}}{\partial \lambda_{e_1}} = 1 - w_1$, $\frac{\partial \lambda_{e_{12}}}{\partial w_1} = -\lambda_{e_1}$
- $\frac{\partial \lambda_{e_{21}}}{\partial \lambda_{e_2}} = w_2$, $\frac{\partial \lambda_{e_{21}}}{\partial w_2} = \lambda_{e_2}$, $\frac{\partial \lambda_{e_{22}}}{\partial \lambda_{e_2}} = 1 - w_2$, $\frac{\partial \lambda_{e_{22}}}{\partial w_2} = -\lambda_{e_2}$
- $\frac{\partial \ell_r}{\partial w_3} = \frac{1}{\delta(1-w_3)} = \frac{1}{\delta e^{-\delta \ell_r}}$.
- $\frac{\partial \gamma_r}{\partial w_4} = 1$.
- The value of the rest partial derivatives in Jacobian is zero.

Therefore, $J = \lambda_{e_1} \lambda_{e_2} \frac{1}{\delta e^{-\delta \ell_r}}$.

We now derive the Hastings ratio.

- Let re be the number of reticulation edges in the new proposed network. The probability of selecting the same edge to remove is $(1/re)$. The probability of choosing Delete-Reticulation operation from the two dimension-changing operations is $1 - \kappa_1$. (κ_1 is the defined in Materials and Methods of the main manuscript).

- To propose adding the reticulation edge, let k be the number of edges in the current network (not the newly sampled one). The probability of adding this edge is $d(1/k)(1/(k-1))(1)(1)$, where the two 1 terms correspond to the uniform density with which the length and inheritance probability of the newly added edge are chosen. If the current network has no reticulations, then $d = 1$, since it is the only one of the two dimension-changing moves that can be performed; otherwise, $d = \kappa_1$.

In summary, the product of the Hastings ratio and $|J|$ for this move is

$$\frac{(1-\kappa_1)(1/re)}{d(1/k)(1/(k-1))}\lambda_{e_1}\lambda_{e_2}\frac{1}{\delta e^{-\delta \ell_r}}.$$

Delete-Reticulation. A reticulation edge $e = (x, y)$ is selected uniformly at random from the set of all reticulation edges in the network and is removed along with its length and inheritance probability. A forced contraction is performed on nodes x and y to remove nodes of in- and out-degree 1. That is, if $e_{11} = (u_1, x)$ and $e_{12} = (x, v_1)$ are edges in the network, then both are removed and replaced by the single edge (u_1, v_1) whose length is $\lambda_{e_{11}} + \lambda_{e_{12}}$. A similar operation is applied to the two edges incoming and outgoing of y. We now derive the Hastings ratio:

- There is probability $1/re$ of selecting the reticulation edge e to remove. The probability of choosing this operation from the two dimension-changing operations is $1 - \kappa_1$.
- The probability of adding the same reticulation to add is

$$d(1/k')(1/(k'-1))(1)(1)$$

  where $d = \kappa_1$ if the the proposed network has at least one reticulation and $d = 1$ if the proposed network has no reticulations. Here, k' is the number of edges in the proposed network (after removing a reticulation edge).

Since the proposal merges four edges into two and removes two parameters (the length and inheritance probabilities) of an edge, the Jacobian can be derived in terms of the reverse proposal:

- $\frac{\partial \lambda_{e_1}}{\partial \lambda_{e_{11}}} = \frac{1}{w_1}$, $\frac{\partial w_1}{\partial \lambda_{e_{11}}} = \frac{1}{\lambda_{e_1}}$, $\frac{\partial \lambda_{e_1}}{\partial \lambda_{e_{12}}} = \frac{1}{1-w_1}$, $\frac{\partial w_1}{\partial \lambda_{e_{12}}} = -\frac{1}{\lambda_{e_1}}$
- $\frac{\partial \lambda_{e_2}}{\partial \lambda_{e_{21}}} = \frac{1}{w_2}$, $\frac{\partial w_2}{\partial \lambda_{e_{21}}} = \frac{1}{\lambda_{e_2}}$, $\frac{\partial \lambda_{e_2}}{\partial \lambda_{e_{22}}} = \frac{1}{1-w_2}$, $\frac{\partial w_2}{\partial \lambda_{e_{22}}} = -\frac{1}{\lambda_{e_2}}$
- $\frac{\partial w_3}{\partial \ell_r} = \delta(1-w_3) = \delta e^{-\delta \ell_r}$.
- $\frac{\partial w_4}{\partial \gamma_r} = 1$.
- The value of the rest partial derivatives in Jacobian is zero.

Therefore, $J = \frac{1}{\lambda_{e_1}\lambda_{e_2}}\delta e^{-\delta \ell_r}$.

In summary, the product of the Hastings ratio and $|J|$ for this move is

$$\frac{d(1/k')(1/(k'-1))}{(1-\kappa_1)(1/re)}\frac{1}{\lambda_{e_1}\lambda_{e_2}}\delta e^{-\delta \ell_r}.$$

1.6 Testing the MCMC sampler

To test our implementation of the sampler, we ran MCMC chains to sample from the prior distribution only; that is, we did not use any data here, so that the likelihood played no role. We ran two experiments:

- Experiment 1: We used the prior on the number of reticulations and branch lengths only.
- Experiment 2: We used the prior on the number of reticulations, branch lengths, and reticulation diameters.

For each of these two experiments, we ran the sampler for 2,020,000 iterations (first 20,000 iterations constitute the burn-in period) and collected 2,000 samples (1,000 iterations per sample) from each chain. Based on these 2,000 sampled, we plotted the distribution of the number of reticulations in the sampled networks. The results of Experiments 1 and 2 are shown in Fig. 3.

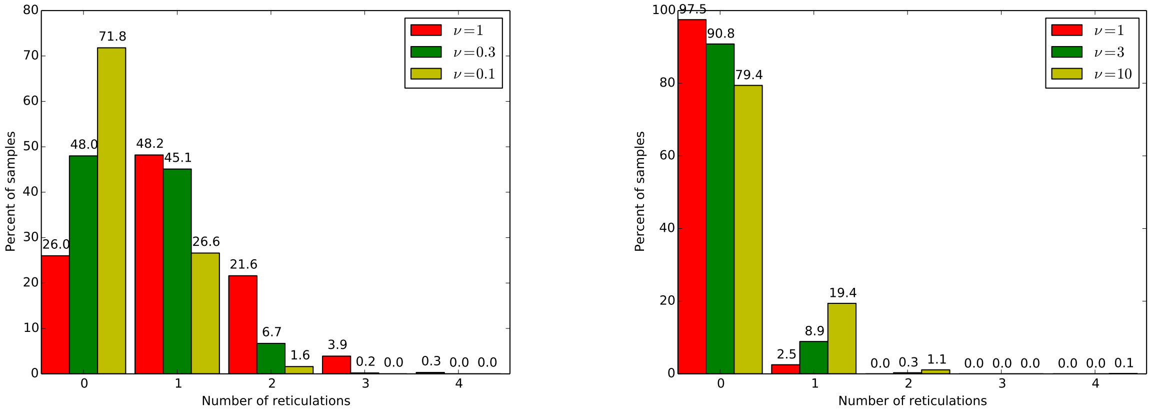

Figure 3. Distribution of the number of reticulations in networks sampled from the posterior when no data is used. Left: Priors on the number of reticulations and branch lengths are used. Right: Priors on the number of reticulations, branch lengths, and reticulation diameter are used. The different bars correspond to the three different Poisson distribution hyperparameters used.

We observe in the left panel of Fig. 3 that as the Poisson prior hyperparameter gets smaller, the number of reticulations in the sampled networks becomes smaller. This is expected since the Poisson distribution on the number of reticulations penalizes adding more reticulations more heavily when its parameter is smaller. For $\nu = 1$, the Poisson prior on 0 and 1 reticulations is equal. However, we observe that significantly more networks with 1 reticulation are sampled than networks with 0 reticulations (i.e., trees). However, in this case, the Hastings ratio plays a bigger role and favors adding the single reticulation. As the sampler go beyond 1 reticulation, the Poisson prior (and the penalty term from the number of networks) cancel out the effect of the Hastings ratio, which is why we observe a decrease again in the frequency of networks with larger numbers of reticulations.

When the prior on the reticulation diameter is added, we observe a significant bias towards trees and networks with single reticulations (the right panel of Fig. 3). The reason is that networks with more reticulations incur an added penalty based on the diameter, which reflects the prior on the reticulation diameter.

1.7 Convergence diagnostics

Mixing evaluation and convergence test are important in MCMC sampling since the samples gathered from a well mixed, converged MCMC chain are more reliable. In this work, we make use of two commonly used diagnostics:

Trace plot. A trace plot is a plot of the iterations versus the sampled value of a variable in an MCMC chain. The variable can be the posterior, the prior, or any other parameters of the distribution. A trace plot would tell us if the chain gets stuck in certain regions of the parameter space, which indicates bad mixing.

95% credible sets from multiple chains. To ensure that results are consistent among chains, we run multiple chains and maintain a 95% credible set of topologies for each chain. We then summarize the frequencies and the posterior probabilities for all topologies in the 95% credible set. Similar results across the chains is desired.

2 Simulations

2.1 Settings for the simulations

Phylogenetic networks. In order to test the performance of our method on varying number of reticulations, branch lengths and inheritance probabilities, we used three phylogenetic networks (see Fig. 4) whose topologies and branch lengths are inspired by a recent work on hybridization in mosquitos [6] and simulated data sets with varying numbers of loci from these three networks. O is the outgroup for rooting gene trees reconstructed from simulated sequences.

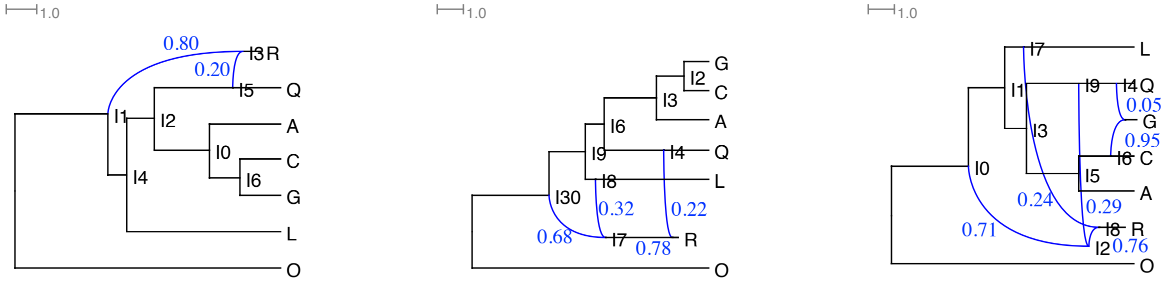

Figure 4. Three phylogenetic networks used to generate simulated data sets. Branch lengths are in coalescent units. The inheritance probabilities are marked in blue.

True gene trees. The program ms [7] was used to simulate gene trees (4 data sets with 128, 320, 800, and 2000 gene trees, respectively) within the branches of each of the phylogenetic networks. The command we used is:

ms 7 numLoci -T -I 7 1 1 1 1 1 1 1 -es 0.28 6 0.8 -ej 0.67 4 3 -ej 0.78 8 1 -ej 1.15 3 2 -ej 2.06 2 1 -ej 2.50 5 1 -ej 2.81 6 1 -ej 4.31 7 1

ms 7 numLoci -T -I 7 1 1 1 1 1 1 1 -es 0.10 3 0.78 -ej 0.48 5 4 -ej 0.87 8 2 -ej 0.99 6 4 -es 1.68 3 0.68 -ej 2.03 4 2 -ej 2.18 9 1 -ej 2.39 2 1 -ej 3.10 3 1 -ej 4.60 7 1

ms 7 numLoci -T -I 7 1 1 1 1 1 1 1 -es 0.21 3 0.95 -ej 0.33 4 8 -ej 0.45 5 3 -es 0.54 1 0.76 -es 0.83 1 0.29 -ej 1.06 6 3 -ej 1.06 1 8 -ej 2.10 3 8 -ej 2.15 2 9 -ej 2.52 8 9 -ej 3.23 9 10 -ej 4.73 7 10

Sequences. We used the simulated gene trees to simulate sequence alignments using the program Seq-gen [8] under the GTR model. The population mutation rate we used is $\theta = 0.036$. The length of sequences is 1000. The command is:

seq-gen -mgtr -s0.018 -f0.2112, 0.2888, 0.2896, 0.2104 -r0.2173, 0.9798, 0.2575, 0.1038, 1, 0.2070 -l1000

where $0.2112, 0.2888, 0.2896, 0.2104$ are the base frequencies of the nucleotides A, C, G and T, respectively, and $0.2173, 0.9798, 0.2575, 0.1038, 1, 0.2070$ are the relative rates of substitutions.

Estimated gene trees. We built 100 bootstrap gene tree topologies for each sequence alignment by RAxML8 [9] under GTR model. The command used was

raxmlHPC-PTHREADS -m GTRGAMMA -# 100 -o O

To assess the difference between the estimated bootstrap trees on each locus and the true tree for that locus, we summed the Robinson-Foulds distance [10] between each bootstrap tree and the true tree, normalized by the number of internal edges in the true tree, and divided the sum by 100. Finally, in each data set, we computed the mean and standard deviation of the normalized Robinson-Foulds distances across all loci in that data set. The results are given in Table 1.

Table 1. The mean and standard deviation of normalized Robinson-Foulds distances between the true gene tree and estimated gene trees for each locus within each simulated data set.

	128	320	800	2000
Data set 1	0.10 ± 0.13	0.11 ± 0.12	0.11 ± 0.12	0.11 ± 0.11
Data set 2	0.11 ± 0.10	0.09 ± 0.10	0.09 ± 0.10	0.09 ± 0.10
Data set 3	0.09 ± 0.11	0.08 ± 0.11	0.09 ± 0.10	0.08 ± 0.10

2.2 Experiments and Results

For each simulated data set, we performed Bayesian inference on the gene trees estimated from the sequence alignments.

MCMC settings.

- Total iterations: 5,050,000.
- Burn-in iterations: 50,000.
- Number of MCMC iterations per sample: 1,000.
- Number of samples sampled from one chain: 5,000.
- Prior: prior on the number of reticulations with Poisson parameter $\nu = 1.0$, exponential priors on branch lengths and reticulation diameters.

Table 2. Total elapsed time (hour) of MCMC chains on the simulate data sets.

	128	320	800	2000
Data set 1	2.77	3.46	4.31	5.02
Data set 2	2.64	3.17	4.78	5.74
Data set 3	2.83	5.06	6.53	9.20

Running times. All MCMC chains were run on NOTS (Night Owls Time-Sharing Service), a batch scheduled HTC cluster running on the Rice Big Research Data (BiRD) cloud infrastructure. We acquired 16 2.6GHz CPU, 1GB RAM per CPU for each task. The running times are given in Table 2.

Note that likelihood computations are the bottleneck in our Bayesian inference method. The likelihood computation times for a given data set are affected by the topology and the branch lengths of the phylogenetic network, the number of distinct gene tree topologies, and the topology of each gene tree. We calculated the mean and standard deviation of the likelihood computation across all distinct gene tree topologies given the true phylogenetic network for each data set. The results are given in Table 3. The large standard deviations clearly indicate the variability in likelihood computation times. In particular, the likelihood computation runtime increases when the number of reticulations in the phylogenetic network gets larger.

Table 3. The mean and standard deviation of the likelihood computation time (ms) for the distinct gene tree topologies within each simulated data set. The number of distinct gene tree topologies are reported in parentheses.

	128 loci	320 loci	800 loci	2000 loci
Data set 1	0.16 ± 0.37 (100)	0.15 ± 0.36 (170)	0.15 ± 0.36 (254)	0.14 ± 0.34 (320)
Data set 2	0.19 ± 0.39 (83)	0.18 ± 0.39 (142)	0.18 ± 0.39 (201)	0.18 ± 0.39 (256)
Data set 3	0.28 ± 0.45 (111)	0.28 ± 0.45 (140)	0.27 ± 0.44 (205)	0.26 ± 0.44 (269)

Results. In total, we ran 12 MCMC chains for the data sets simulated from the three phylogenetic networks and varying numbers of loci 128, 320, 800, and 2000. The trace plots are shown in Fig. 5.

Furthermore, we summarized the networks in the 95% credible sets. The results are shown in Fig. 6.

In particular, the results were as follows:

- For the true phylogenetic network with 1 reticulation node,
  - regardless of the number of loci used, all the 4 MCMC chains had a 95% credible set consisting of the true topology (1-reticulation) only (Fig. 6A).
- For the true phylogenetic network with 2 reticulation nodes,
  - on 128 and 320 loci, both MCMC chains had a 95% credible set consisting of the 1-reticulation topology only (Fig. 6A).
  - on 800 and 2000 loci, both MCMC chains had a 95% credible set consisting of the true topology (2-reticulation) only (Fig. 6B).
- For the true phylogenetic network with 3 reticulation nodes,

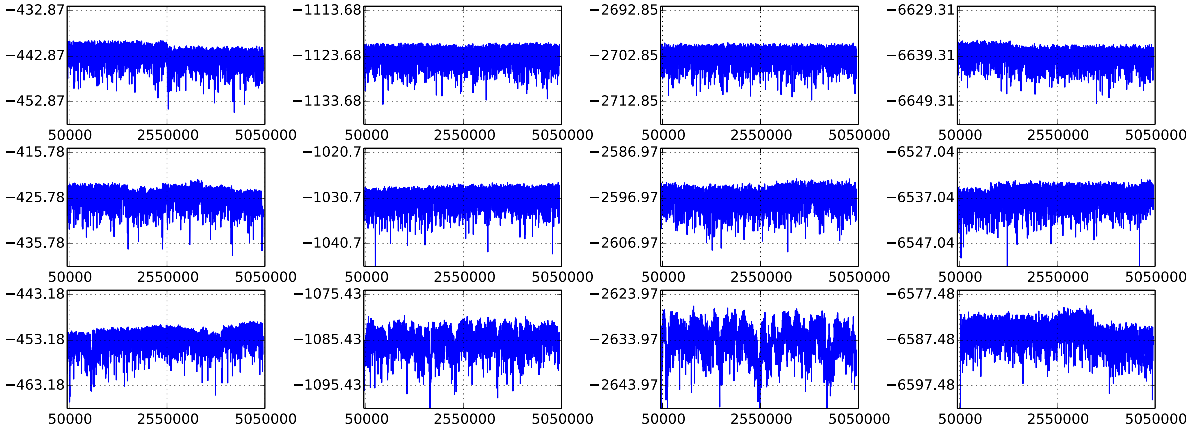

Figure 5. Trace plots of the MCMC samples from the simulated data sets. Rows from top to bottom correspond to data sets generated on the networks with 1, 2, and 3 reticulations, respectively. The columns from left to right correspond to data sets with 128, 320, 800, and 2000 gene trees, respectively. The burn-in iterations are excluded.

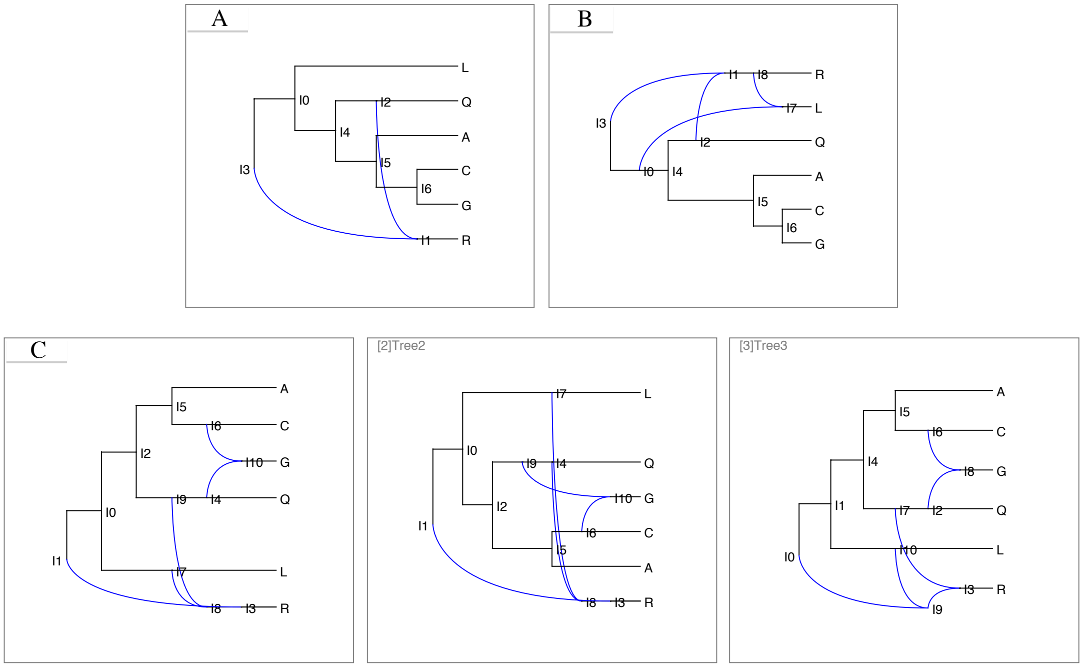

Figure 6. The phylogenetic network topologies in the 95% credible sets of the results using the simulated data.

– on 128 loci, the MCMC chain had a 95% credible set consisting of the 1-reticulation topology only (Fig. 6A).

– on 320 loci, the MCMC chain had a 95% credible set consisting of the 2-reticulation topology

only (Fig. 6B).

– on 800 and 2000 loci, the MCMC chain had a 95% credible set consisting of three 3-reticulation topologies (Fig. 6C). Note that these topologies are indistinguishable using the gene tree topologies and employing our likelihood formulation, and thus can be viewed as equivalent to the true topology.

To summarize, the method shows very good performance on these simulated data sets.

3 Analysis of a bread wheat (*Triticum aestivum*) data set

Marcussen *et al.* [11] investigated ancient hybridization among the ancestral genomes of bread wheat by performing parsimonious inference of hybridization in the presence of ILS [12] implemented in PhyloNet [13]. 2269 gene trees were constructed from three subgenomes of wheat TaA (*T. aestivum* A subgenome), TaB (*T. aestivum* B subgenome), TaD (*T. aestivum* D subgenome). Using this data set, they inferred a species phylogeny, shown in Fig. 7. We reanalyzed this data set using our newly developed method.

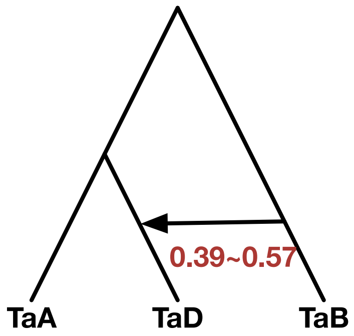

Figure 7. The species phylogeny reported in [11] and inferred from a data set of 2269 gene trees using parsimonious inference of hybridization in the presence of ILS implemented in PhyloNet [13].

3.1 Data preprocessing

We downloaded the sequence alignments of 2269 genes from Dryad Digital Repository (doi:10.5061/dryad.f6c34). Each alignment is composed of genes from TaA, TaB, TaD and three outgroups Bd (*Brachypodium distachyon*, Os (*Oryza sativa*) and Hv (*Hordeum vulgare*).

We built 100 bootstrap gene tree topologies for each alignment using RAxML8 [9] under GTR model. The command is

raxmlHPC-PTHREADS -m GTRGAMMA -# 100 -o Outgroup

3.2 MCMC settings

- Total iterations: 5,050,000.
- Burn-in iterations: 50,000.
- Number of MCMC iterations per sample: 1,000.
- Number of samples in one chain: 5,000.

- Prior: prior on the number of reticulations (Poisson parameter $\nu = 1.0$), prior on branch lengths, prior on reticulation diameter.

3.3 Runtime.

The elapsed time for this analysis is 2.20 hr.

3.4 Results

The three topologies composing the 95% credible set are shown in Fig. 8.

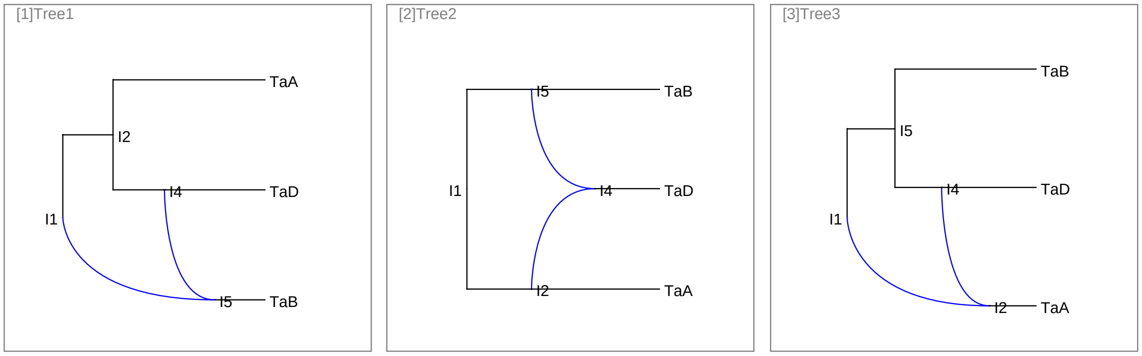

Figure 8. The three topologies composing the 95% credible set for the wheat data set.

The trace plot is displayed in Fig. 9.

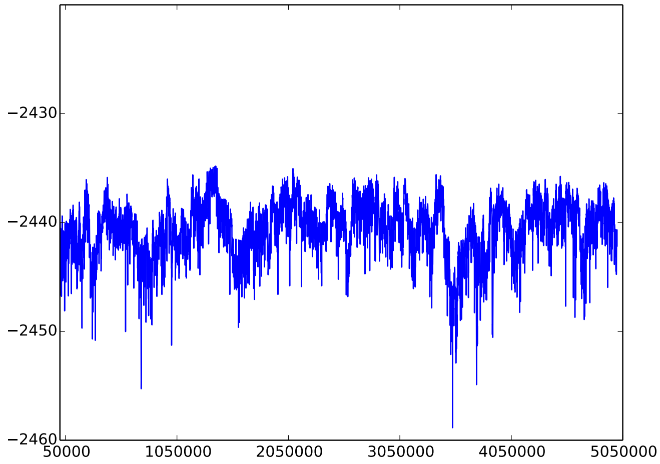

Figure 9. Trace plot of the MCMC samples from the bread wheat data set. The burn-in iterations are excluded.

3.5 Gene trees with branch lengths

Fig. 10 shows data on pairwise distances inferred from across all loci for all pairs of taxa. Since the smallest pairwise distance per pair of taxa is a upper bound on the speciation time of these taxa (according to the likelihood formulation used here), it is clear that using gene tree branch lengths in the inference step would bias the inferred network and produce erroneous results. A similar observation was observation was made in [14].

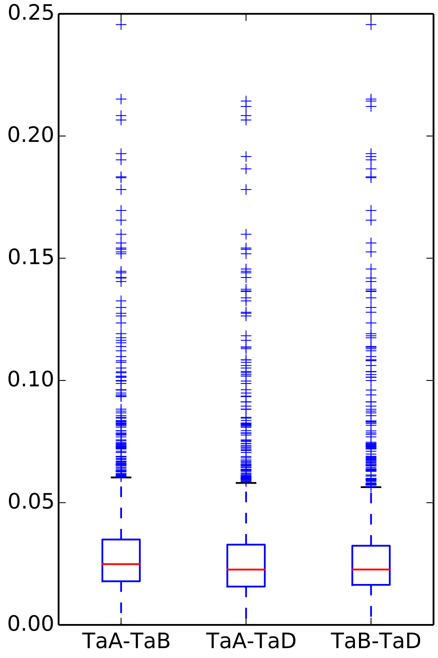

Figure 10. Whisker-box plot of all pairwise distances for each pair of species in the wheat data set. Gene trees and their branch lengths were estimated using maximum likelihood in PAUP*. A pairwise distance between two leaves in a gene tree is the sum of branch lengths on the path between the two leaves in the tree. The points on the x-axis correspond to all possible pairs of taxa, and the various pairwise distances per pair come from the loci that were used in the inference.

4 Analysis of Anopheles mosquitoes (*An. gambiae* complex) data set

Fontaine et al. recently reported on hybridization and extensive introgression in the *An. gambiae* complex [6]. The authors constructed a phylogenetic network by adding three major reticulation edges to the species tree constructed from X chromosome. They used gene tree analysis to detect the location of the reticulation edges. More recently, Wen *et al.* [15] applied systematic inference of phylogenetic networks to the data using the maximum likelihood method of [3]. They provided an a new view on the evolutionary history of the species. We reanalyzed the same data used in [15].

4.1 Data preprocessing

We downloaded the MAF genome alignment from high depth field samples from Dryad (doi:10.5061/dryad.f4114). The species we included in our analysis are *An. gambiae* (G), *An. coluzzii* (C), *An. arabiensis* (A), *An. quadriannulatus* (Q), *An. merus* (R) and *An. melas* (L). *An. christyi* serves as the outgroup for gene tree reconstruction and rooting. For each chromosome (2L, 2R, 3L 3R, X), we randomly sampled genome alignment chunks from the original dataset. The alignment chunks are at lease 64 kb far away from each other to minimize the likelihood of dependence among loci.

The total number of alignments we sampled for each chromosome is

2L	2R	3L	3R	X	all
669	849	564	709	228	3019

We built 100 bootstrap trees (topology only) for each alignment using RAxML8 [9] under GTR model. The command we used is

raxmlHPC-PTHREADS -m GTRGAMMA -# 100 -o Outgroup

4.2 MCMC settings for inference from the sex chromosome and autosomes

We separate the inference from the sex chromosome and autosomes since the effective population sizes differ between sex chromosomes and autosomes.

The settings for the MCMC chain used in inference is as follows.

- Total iterations: 5,050,000
- Burn-in iterations: 50,000
- Number of MCMC iterations per sample: 1,000
- Number of samples: 5,000
- Prior: prior on the number of reticulations, prior on branch lengths, prior on diameters of hybridizations

For the X chromosome, we used Poisson parameter $\nu = 1.0$. For the autosome data, we tried three different values, $\nu = 0.1, 1.0, 10.0$, in order to test the effect of the prior.

4.3 Results from the X chromosome data set

Only one topology exists in the 95% credible sets (Fig. 11).

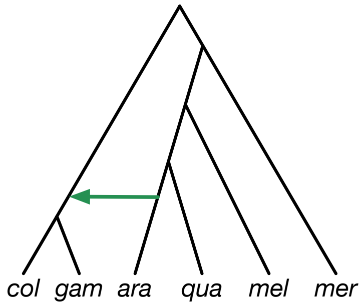

Figure 11. The single topology in the 95% credible set from the mosquito X chromosome data set.

The trace plot on the posterior distribution of the MCMC chain is displayed in Fig. 12. The elapsed time is 7.65 hr.

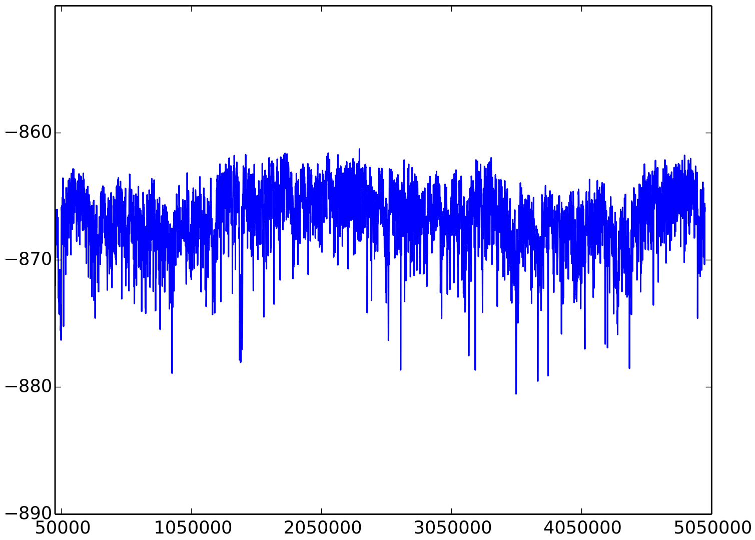

Figure 12. Trace plot of the MCMC samples from the mosquito X chromosome data set. The burn-in iterations are excluded.

4.4 Results from the autosome data set

Aside from reanalyzing the autosome dataset, we also studies the effect of priors on different data sizes. We sampled 311 and 931 loci from the full data set (2791 loci), and then performed Bayesian inference on different priors (Poisson parameter $\nu = 0.1, 1, 10$):

- On 311 loci,
  - for $\nu = 0.1$, only one topology exists in the 95% credible set (Fig. 13[1]), which is a network with one reticulation node.
  - for $\nu = 1.0$, the 95% credible set contains 4 topologies (Fig. 13[1]-[4]). The proportions of the 1-reticulation and 2-reticulation topologies are 75.3% and 22.5%, respectively.
  - for $\nu = 10$, the 95% credible set contains 4 topologies (Fig. 13[1]-[4]). The proportions of the 1-reticulation and 2-reticulation topologies are 27.7% and 63.2%, respectively.

  By increasing ν, the proportion of 2-reticulation topologies are increased.
- On 931 loci, varying ν did not affect the results—the 95% credible set contains 3 indistinguishable, 2-reticulation networks (Fig. 13[2]-[4]).
- On 2,791, varying ν did not affect the results—the 95% credible set contains 3 indistinguishable, 3-reticulation networks (Fig. 14).

These results demonstrate that when the data size is small, the prior could play an important role in the posterior distribution, and when the data size gets larger, varying the prior (the Poisson parameter in our case) could hardly affect the results. This further attests to the good performance of our method.

The trace plot for the case of $\nu = 1.0$ and 2791 loci is shown in Fig. 15.

The runtimes are given in Table 4. We can see that when the data size and the expected number of reticulations (reflected by ν) are smaller, the sampling space is almost within the 1-reticulation network

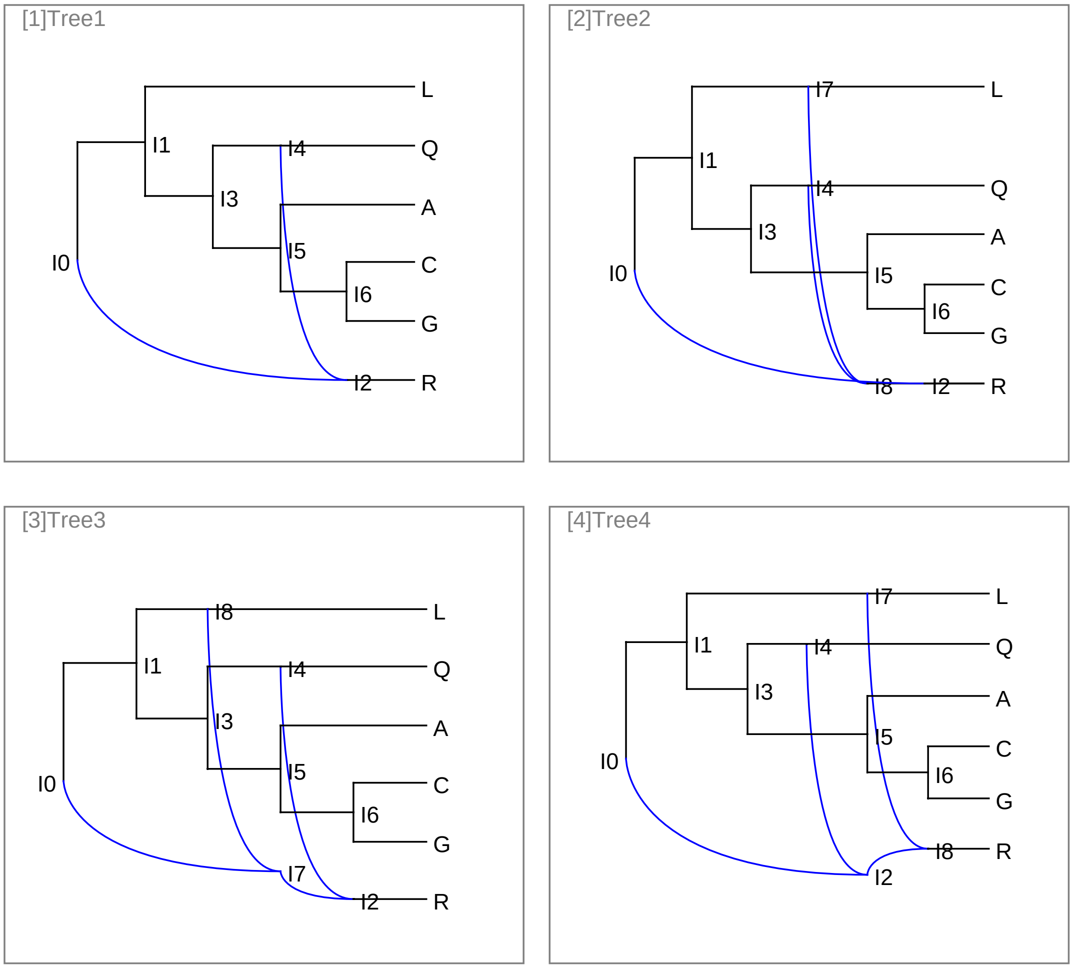

Figure 13. The topologies in the 95% credible sets sampled from the 311 and 931 autosome loci. Note that the topologies [2]-[4] are indistinguishable using gene tree topologies under the likelihood function used here.

space and the likelihood computation is much faster. When the data size and the expected number of reticulations gets larger, the likelihood computation is more time demanding, thus the total running time is longer.

Table 4. Total elapsed time (hour) of MCMC chains of the mosquito data analyses.

	$\nu = 0.1$	$\nu = 1$	$\nu = 10$
311 loci	9.02	8.45	12.02
931 loci	12.48	13.56	15.50
2791 loci	23.71	24.15	29.78

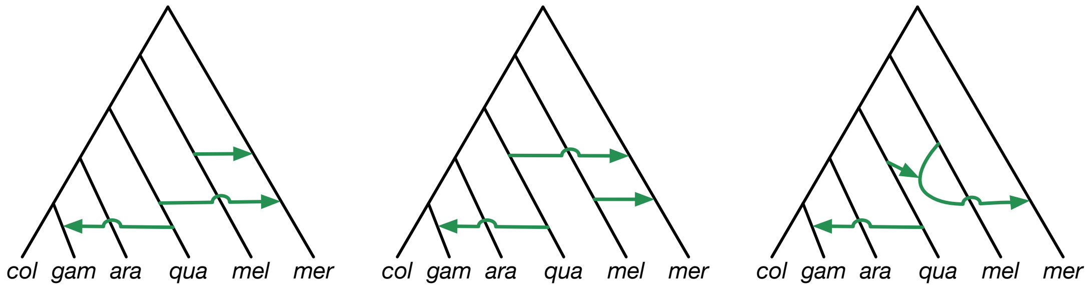

Figure 14. The topologies in the 95% credible sets sampled from the full autosome data set. Note that the topologies are indistinguishable using gene tree topologies under the likelihood function used here.

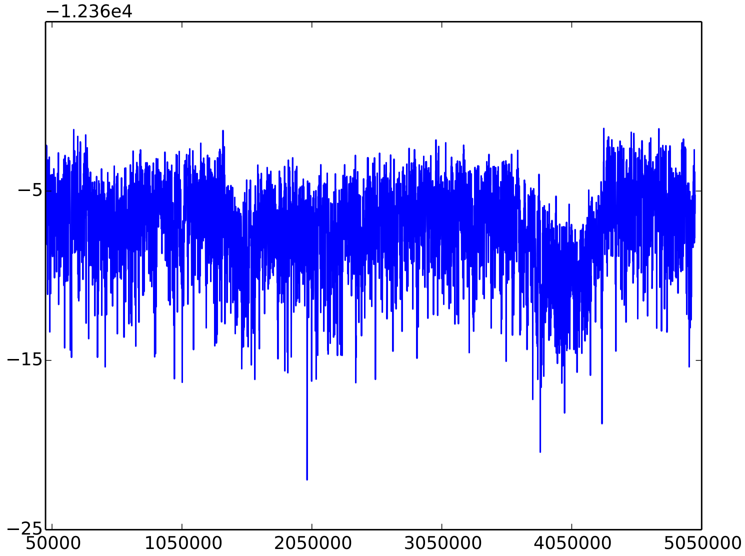

Figure 15. Trace plot of the MCMC samples from the mosquito autosome data set. The number of loci is 2791 and the Poisson hyperparameter value is $\nu = 1.0$. The burn-in iterations are excluded.

4.5 Gene trees with branch lengths

Fig. 16 shows data on pairwise distances inferred from across all loci for all pairs of taxa. Results are similar to those we observed above in the wheat data set and would have similar implications on inference from gene trees with branch lengths.

5 Analysis of a house mouse (*Mus musculus*) data set

The house mouse data set is composed of individuals sampled from five populations: *M. m. domesticus* from France (DF), *M. m. domesticus* from Germany (DG), *M. m. musculus* from the Czech Republic (MZ), *M. m. musculus* from Kazakhstan (MK), and *M. m. musculus* from China (MC). To satisfy the assumption of free recombination between loci, local phylogenies were sampled at 100 kb intervals. Local

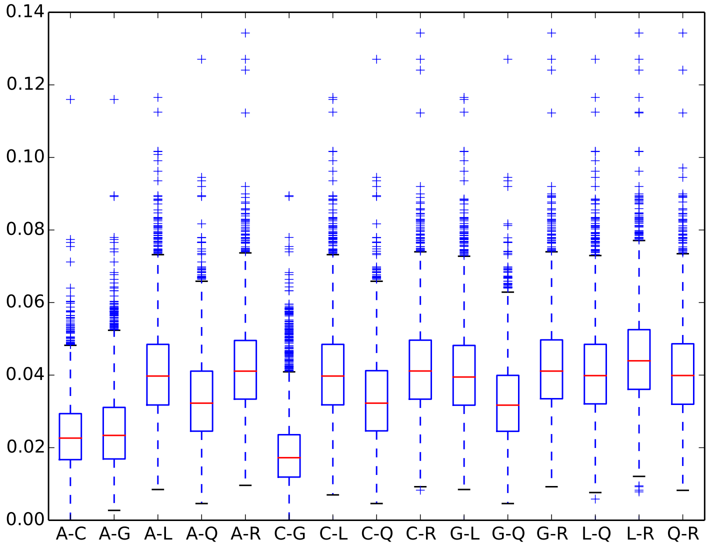

Figure 16. Whisker-box plot of all pairwise distances for each pair of species in the mosquito data set. Gene trees and their branch lengths were estimated using maximum likelihood in PAUP*. A pairwise distance between two leaves in a gene tree is the sum of branch lengths on the path between the two leaves in the tree. The points on the x-axis correspond to all possible pairs of taxa, and the various pairwise distances per pair come from the loci that were used in the inference.

phylogenies were rooted using *R. norvegicus* as an outgroup. In total, 20,639 local phylogenies were reconstructed.

Yu *et al.* [3] investigated the house mouse dataset using maximum likelihood and reported two main hybridization events. We reanalyzed the house mouse data set using our new Bayesian inference method.

5.1 Data Preprocessing

In the preliminary analysis, we found several 4-reticulation networks with hybridization near the root (between the two branches emanating from the root), which indicates poor signal in the data. This is not surprising, given that this data set differs from the other two in that it consists of individuals of the same species, rather than different species.

Since for each locus we inferred 100 bootstrap trees, we computed the majority-rule consensus of the 100 trees for each locus, and we analyzed the number of loci that have gene trees with 0-3 internal branches to assess the signal in the data. Gene trees with 0 internal branches are star phylogenies with no signal at all. Gene trees with 3 internal branches are fully resolved.

We found that 11,457 (55.5%) loci have gene trees with 3 internal branches. Among the 11,457 gene trees built on these loci, 98 distinct topologies were present. Note that for 5 taxa, there are only 105 possible gene tree topologies. In other words, for almost every fully-resolved gene tree there are some loci that support it. The distribution of the number of fully-resolved gene trees that are supported by different numbers of loci is given in

The distribution shows that three distinct gene tree topologies are supported by over 2000 loci each. On the other end of the distribution, 45 distinct gene trees are supported by between 1 and 9 loci only.

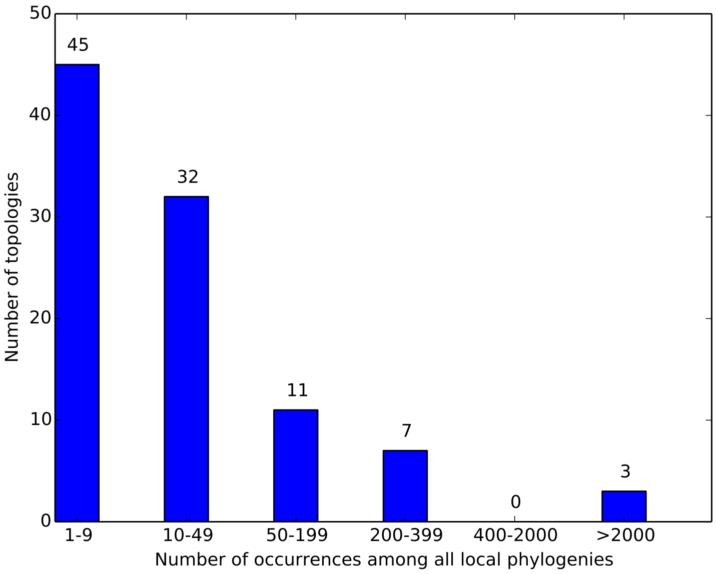

Figure 17. Distribution of topologies on the number of occurrences.

To capture the main hybridization events, we performed the inference on the fully resolved 21 gene tree topologies that are supported by at least 50 loci each. This reduced the size of the data set from 20,639 gene trees to 10,575 gene trees.

5.2 Settings

We employed Metropolis-coupled MCMC (MC^3) (described in [16]) to help the sampler traverse the posterior landscape.

The settings for MCMC inference are

- Total iterations: 4,050,000
- Burn-in iterations: 50,000
- Number of MCMC iterations per sample: 1,000
- Number of samples: 4,000
- Prior Parameter: Poisson parameter $\nu = 1.0$)
- (MC^3) Number of chains: 3 (1 cold chain, 2 heated chains)
- (MC^3) Temperature settings: 1 (for cold chain), 2, 4
- (MC^3) Swap frequency: swap two random chains once every 100 iteration
- (MC^3) Starting tree for each chain: a random tree from the input phylogenies

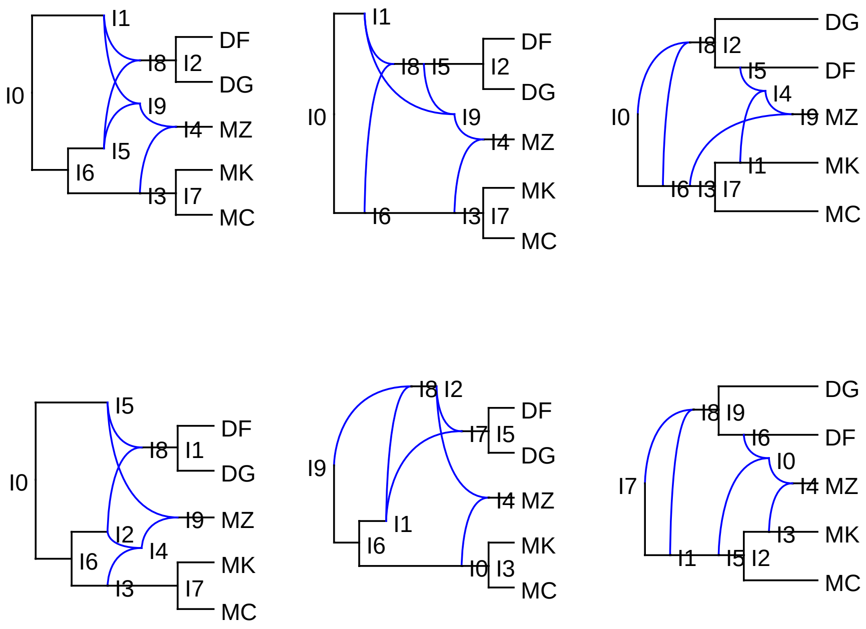

Figure 18. The top six topologies sampled in the 95% credible set from mouse data set.

5.3 Results

The topologies in the 95% credible set are shown in Fig. 18. Unlike the cases of the wheat and mosquito data sets, where multiple indistinguishable networks were sampled, several distinguishable (population) networks with similar posterior values were sampled from the mouse dataset. This issue emphasizes the applicability of the method to population data and, at the same time, the complexity in the inferred evolutionary history in this case due to extensive gene flow.

The trace plot is shown in Fig. 19.

The elapsed time is 44.65 hr, which is longer than the other datasets because we ran two heated chains along with the cold chain.

5.4 Gene trees with branch lengths

Fig. 20 shows data on pairwise distances inferred from across all loci for all pairs of taxa. Results are similar to those we observed above in the wheat data set and would have similar implications on inference from gene trees with branch lengths. In this case, however, the data consists of multiple individuals of the same species. That is, this is a data set of very low divergence levels. This is clearly obvious from the pairwise distances, and further highlights the complexity of analyzing such a data set, despite the applicability of the method.

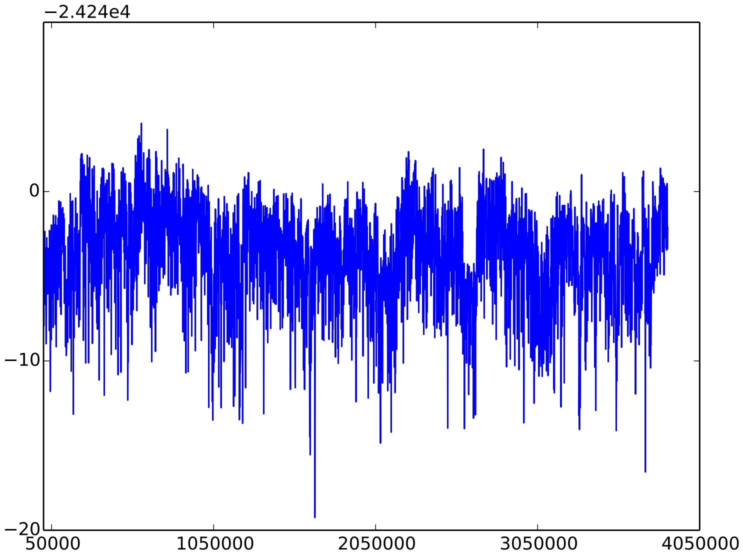

Figure 19. Trace plot of the MCMC samples from the mouse data set. The burn-in iterations are excluded.

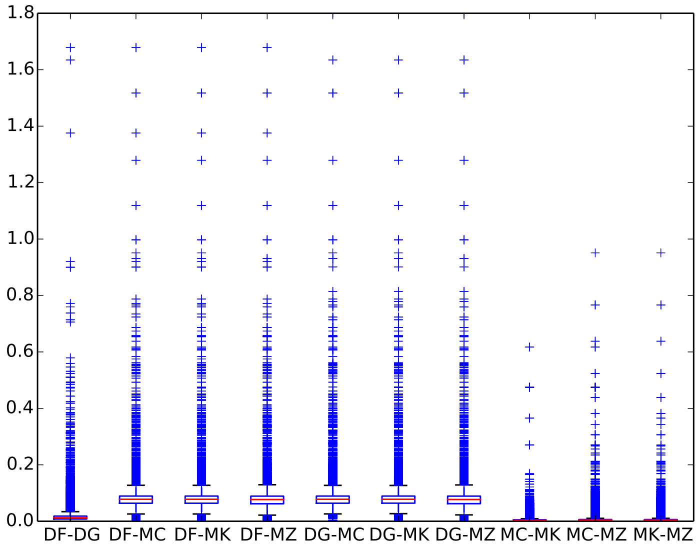

Figure 20. Whisker-box plot of all pairwise distances for each pair of species in the mouse data set. Gene trees and their branch lengths were estimated using maximum likelihood in PAUP*. A pairwise distance between two leaves in a gene tree is the sum of branch lengths on the path between the two leaves in the tree. The points on the x-axis correspond to all possible pairs of taxa, and the various pairwise distances per pair come from the loci that were used in the inference.

References

1. Yu Y, Degnan JH, Nakhleh L (2012) The probability of a gene tree topology within a phylogenetic network with applications to hybridization detection. PLoS genetics 8: e1002660.

2. Yu Y, Ristic N, Nakhleh L (2013) Fast algorithms and heuristics for phylogenomics under ILS and hybridization. BMC Bioinformatics 14: S6.

3. Yu Y, Dong J, Liu KJ, Nakhleh L (2014) Maximum likelihood inference of reticulate evolutionary histories. Proceedings of the National Academy of Sciences 111: 16448–16453.

4. Bloomquist E, Suchard M (2010) Unifying vertical and nonvertical evolution: A stochastic ARG-based framework. Systematic Biology 59: 27-41.

5. Lewis PO, Holder MT, Holsinger KE (2005) Polytomies and Bayesian phylogenetic inference. Systematic Biology 54: 241–253.

6. Fontaine MC, Pease JB, Steele A, Waterhouse RM, Neafsey DE, et al. (2015) Extensive introgression in a malaria vector species complex revealed by phylogenomics. Science 347: 1258524.

7. Hudson R (2002) Generating samples under a Wright-Fisher neutral model of genetic variation. Bioinformatics 18: 337-338.

8. Rambaut A, Grassly NC (1997) Seq-gen: An application for the Monte Carlo simulation of DNA sequence evolution along phylogenetic trees. Comp Appl Biosci 13: 235-238.

9. Stamatakis A (2014) RAxML version 8: a tool for phylogenetic analysis and post-analysis of large phylogenies. Bioinformatics 30: 1312–1313.

10. Robinson D, Foulds L (1981) Comparison of phylogenetic trees. Math Biosci 53: 131–147.

11. Marcussen T, Sandve SR, Heier L, Spannagl M, Pfeifer M, et al. (2014) Ancient hybridizations among the ancestral genomes of bread wheat. Science 345: 1250092.

12. Yu Y, Barnett RM, Nakhleh L (2013) Parsimonious inference of hybridization in the presence of incomplete lineage sorting. Systematic Biology 62: 738-751.

13. Than C, Ruths D, Nakhleh L (2008) PhyloNet: a software package for analyzing and reconstructing reticulate evolutionary relationships. BMC bioinformatics 9: 322.

14. DeGiorgio M, Degnan JH (2014) Robustness to divergence time underestimation when inferring species trees from estimated gene trees. Systematic biology 63: 66–82.

15. Wen D, Yu Y, Hahn MW, Nakhleh L (2016) Reticulate evolutionary history and extensive introgression in mosquito species revealed by phylogenetic network analysis. Molecular Ecology .

16. Altekar G, Dwarkadas S, Huelsenbeck JP, Ronquist F (2004) Parallel Metropolis coupled Markov chain Monte Carlo for Bayesian phylogenetic inference. Bioinformatics 20: 407–415.
